# Targeting the *PTTG1* oncogene impairs proliferation and invasiveness of melanoma cells sensitive or with acquired resistance to the BRAF inhibitor dabrafenib

**DOI:** 10.18632/oncotarget.23052

**Published:** 2017-12-09

**Authors:** Simona Caporali, Ester Alvino, Pedro Miguel Lacal, Federica Ruffini, Lauretta Levati, Laura Bonmassar, Alessandro Scoppola, Paolo Marchetti, Simona Mastroeni, Gian Carlo Antonini Cappellini, Stefania D’Atri

**Affiliations:** ^1^ Laboratory of Molecular Oncology, Istituto Dermopatico dell’Immacolata-IRCCS, Rome, Italy; ^2^ Institute of Translational Pharmacology, National Council of Research, Rome, Italy; ^3^ Department of Oncology and Dermatological Oncology, Istituto Dermopatico dell’Immacolata-IRCCS, Rome, Italy; ^4^ UOC Oncologia, University of Rome “La Sapienza”, Rome, Italy; ^5^ Clinical Epidemiology Unit, Istituto Dermopatico dell’Immacolata-IRCCS, Rome, Italy

**Keywords:** melanoma, proliferation, invasion, PTTG1, dabrafenib

## Abstract

The *pituitary tumor transforming gene 1* (*PTTG1*) is implicated in tumor growth, metastasis and drug resistance. Here, we investigated the involvement of *PTTG1* in melanoma cell proliferation, invasiveness and response to the BRAF inhibitor (BRAFi) dabrafenib. We also preliminary assessed the potential value of circulating PTTG1 protein to monitor melanoma patient response to BRAFi or to dabrafenib plus trametinib. Dabrafenib-resistant cell lines (A375R and SK-Mel28R) were more invasive than their drug-sensitive counterparts (A375 and SK-Mel28), but expressed comparable PTTG1 levels. Dabrafenib abrogated PTTG1 expression and impaired invasion of the extracellular matrix (ECM) in A375 and SK-Mel28 cells. In contrast, it affected neither PTTG1 expression in A375R and SK-Mel28R cells, nor ECM invasion in the latter cells, while further stimulated A375R cell invasiveness. Assessment of proliferation and ECM invasion in control and *PTTG1*-silenced A375 and SK-Mel28 cells, exposed or not to dabrafenib, demonstrated that the inhibitory effects of this drug were, at least in part, dependent on its ability to down-regulate PTTG1 expression. *PTTG1*-silencing also impaired proliferation and invasiveness of A375R and SK-Mel28R cells, and counteracted dabrafenib-induced stimulation of ECM invasion in A375R cells. Further experiments performed in A375R cells indicated that *PTTG1*-silencing impaired cell invasiveness through inhibition of MMP-9 and that PTTG1 expression and ECM invasion could be also reduced by the CDK4/6 inhibitor LEE011. PTTG1 targeting might, therefore, represent a useful strategy to impair proliferation and metastasis of melanomas resistant to BRAFi. Circulating PTTG1 also appeared to deserve further investigation as biomarker to monitor patient response to targeted therapy.

## INTRODUCTION

The pituitary tumor transforming gene 1 (*PTTG1*), codes for a multifunctional protein involved in a variety of cellular processes [reviewed in 1–4]. As a vertebrate securin, the PTTG1 protein plays a crucial role in the regulation of sister chromatid separation during mitosis [1–4]. In addition, it participates in DNA repair, apoptosis, senescence, metabolism and gene transcription [1–4].

*PTTG1* is considered an oncogene [5, 6], and it is over-expressed in a variety of cancer cell lines as well as in a wide range of primary and metastatic tumors [1–4, 7–12], including melanoma [13]. Notably, *PTTG1* is one of the 17 gene-expression signature predicting metastasis and shorter survival in multiple tumor types [14] and it is among the top-20 genes whose elevated expression was found to be associated with metastatic dissemination of melanoma [15, 16]. The involvement of PTTG1 in tumor growth and metastasis is further highlighted by several studies showing that in cancer cell lines of various histological derivation ectopic expression of *PTTG1* enhanced proliferation and/or invasiveness, whereas *PTTG1* silencing produced opposite results [7, 8, 10–12, 17–22].

Multiple molecular mechanisms appear to underlie the growth and invasion promoting activity of PTTG1. For instance, Yoon *et al.* [7] demonstrated that in breast cancer cells PTTG1 promotes epithelial to mesenchymal transition (EMT) and expansion of the cancer stem cell population via AKT activation, while Zhang *et al.* [17] reported that PTTG1 enhanced breast cancer cell proliferation through inhibition of TGF-β signaling. PTTG1 can also affect the invasive capacity of cancer cells through positive modulation of several matrix metalloproteinases (MMPs) [8, 10, 18, 21].

A number of experimental evidences also support a role of PTTG1 in the regulation of cancer cell response to therapy. PTTG1 interacts with p53 and negatively modulates p53-mediated transcriptional activity and apoptosis [23]. On the other hand, p53 was shown to directly repress *PTTG1* transcription, and this molecular event was suggested to contribute to apoptosis induced by p53 up-regulation in colon cancer cells treated with 5-fluorouracil [24]. PTTG1 loss was also demonstrated to increase colon cancer cell sensitivity to ionizing radiation, adriamycin, doxorubicin or Trichostatin A [25, 26]. In breast cancer, *PTTG1* was among the eight genes significantly overexpressed in tumor specimens of patients who relapsed on tamoxifen treatment as compared with tumor of patients who did not [27]. Furthermore, high levels of PTTG1 were found to promote resistance to gefitinib-induced apoptosis in various tumor cell lines [28] and to be associated with saracatinib resistance in ovarian cancer cells [29].

Although *PTTG1* is over-expressed in melanoma specimens [13] and is included in the gene panel identifying a metastatic behavior in this tumor [15, 16], no data are available on the biological activity of the PTTG1 protein in melanoma cells, with exception of a previous study by our group [30]. In that investigation we showed that *PTTG1* silencing inhibited proliferation of melanoma cells and that the growth suppressive effects of the cyclin-dependent kinase (CDK) inhibitor PHA-848125 was in part dependent on drug-induced down-regulation of PTTG1.

In the present study, we investigated the role of *PTTG1* in melanoma cell proliferation, invasiveness and response to the BRAF inhibitor (BRAFi) dabrafenib by using two pairs of syngeneic melanoma cell lines sensitive or with acquired resistance to the drug. Moreover, based on our results, we assessed whether changes of PTTG1 plasma levels occur in melanoma patients subjected to therapy with BRAFi or the combination of dabrafenib plus the MEK inhibitor (MEKi) trametinib.

## RESULTS

### Generation and characterization of the SK-Mel28R subline with acquired resistance to dabrafenib

We previously reported that the dabrafenib-resistant A375R cell line was more invasive and secreted higher levels of VEGF-A and MMP-9 as compared with the parental A375 cell line [31]. We also showed that exposure to dabrafenib reduced invasiveness and VEGF-A secretion in A375 cells, whereas it increased invasiveness, VEGF-A and MMP-9 release in A375R cells [31].

In the present study, we generated an additional dabrafenib-resistant cell line, (i.e. SK-Mel28R), that was compared to its parental cell line (i.e. SK-Mel28) for the ability to invade the extracellular matrix (ECM), under basal condition and in response to exogenously added VEGF-A, as well as for VEGF-A and MMP-9 secretion. The effects of dabrafenib treatment on these cellular processes were also investigated in both cell lines.

**Figure 1:**
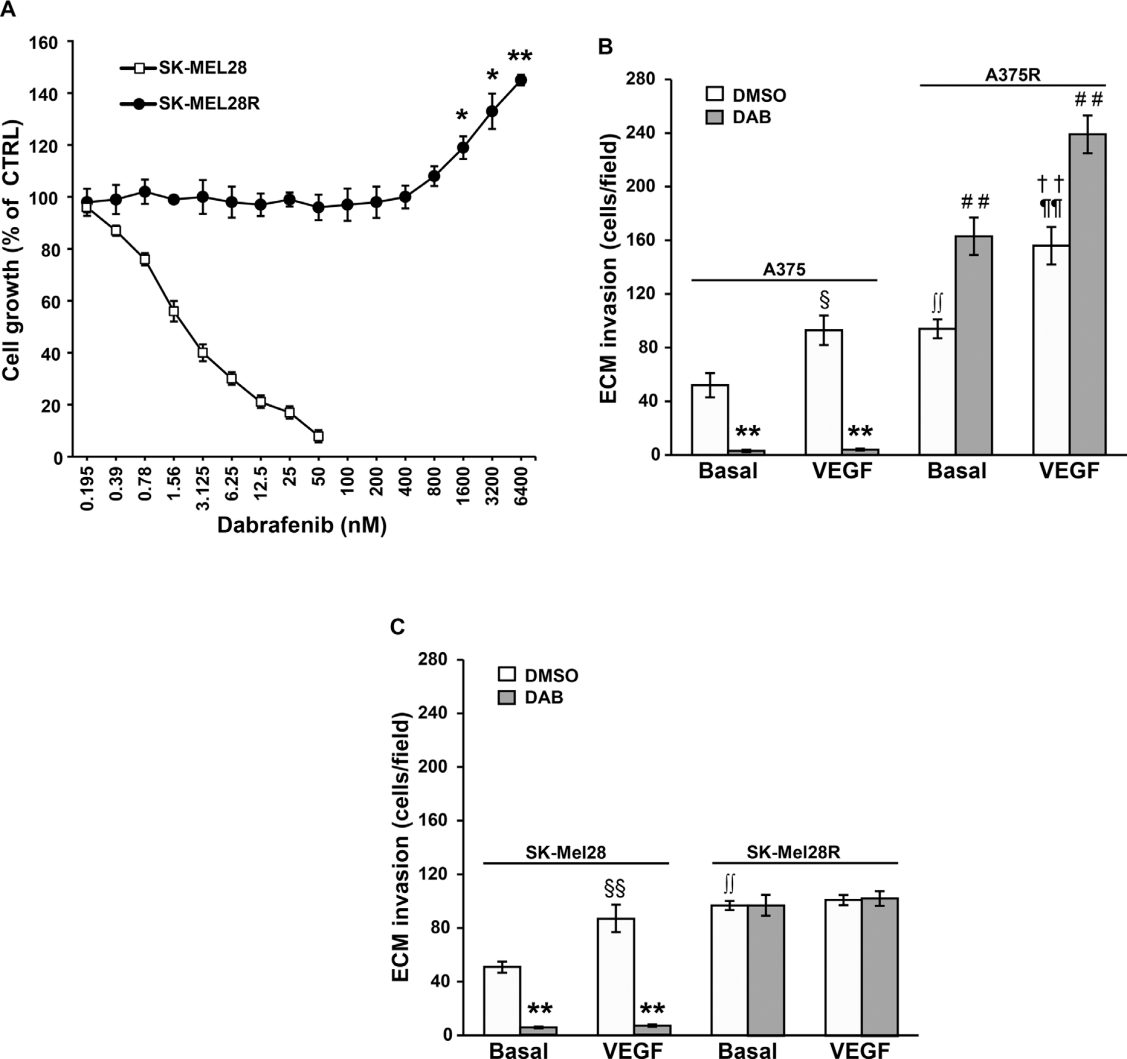
Characterization of the dabrafenib-resistant SK-Mel28R cells (**A**) SK-Mel28 and SK-Mel28R cells were incubated with graded concentrations of dabrafenib or with DMSO alone for five days and then proliferation was assessed by the MTT assay. Data are expressed in terms of percentage of growth of cells treated with dabrafenib with respect to cells treated with DMSO alone (CTRL). Each value represents the arithmetic mean of four (SK-Mel28) or three (SK-Mel28R) independent experiments. Bars, standard error of the mean (SEM). For each experiment, the dabrafenib IC_50_ value was calculated as described in the “Materials and methods” section. The IC_50_ mean value ± SEM was 3.05 ± 0.39 nM for SK-Mel28 cells and not assessable for SK-Mel28R cells. ^**^P < 0.01 and ^*^P < 0.05 comparing dabrafenib-treated with DMSO-treated SK-Mel28R cells. (**B**) A375 and A375R cells were cultured in the presence of 100 nM dabrafenib (DAB) or DMSO alone for 48 h. Thereafter, cell ability to invade the ECM, either spontaneously (Basal) or in response to VEGF-A was evaluated. Data are expressed as number of invaded cells per microscopic field. Each value represents the arithmetic mean ± SEM of four independent experiments. ^§^*P* < 0.05 A375/VEGF-A *versus* (*vs*) A375/Basal; ^**^P < 0.01 A375/DAB *vs* A375/DMSO; ^††^*P* < 0.01 A375R/VEGF-A *vs* A375R/Basal; ^##^*P* < 0.01 A375R/DAB *vs* A375R/DMSO; ^**∫∫**^*P* < 0.01 A375R/basal *vs* A375/basal; ^¶¶^*P* < 0.01 A375R/VEGF-A *vs* A375/VEGF-A. (**C**) SK-Mel28 and SK-Mel28R were treated and analyzed for ECM invasion as described in (B). Data are expressed as number of invaded cells per microscopic field. Each value represents the arithmetic mean ± SEM of six (SK-Mel28) or four (SK-Mel28R) independent experiments. ^§§^*P* < 0.01 SK-Mel28/VEGF-A *vs* SK-Mel28/Basal; ^**^P < 0.01 SK-Mel28/DAB *vs* SK-Mel28/DMSO; ^**∫∫**^*P* < 0.01 SK-Mel28R/basal *vs* SK-Mel28/basal.

MTT assays, performed after five days of cell culture with graded concentrations of dabrafenib, confirmed that SK-Mel28 cells were highly susceptible to the growth suppressive effects of dabrafenib, even though the drug IC_50_ value was about 3-fold higher than that previously observed in A375 cells [31]. In contrast, proliferation of SK-Mel28R cells was not affected by drug concentrations up to 800 nM, and even stimulated by drug concentrations ranging between 1600 nM and 6400 nM (Figure 1A). In agreement with the results obtained with A375 and A375R cell lines, which were included in the invasion assays for comparison (Figure 1B), SK-Mel-28R cells were about 2-fold more invasive than the corresponding dabrafenib-sensitive parental cells (Figure 1C). However, while exposure to VEGF-A caused an increase of ECM invasion in both A375 and A375R cells, as previously reported [31], only SK-Mel28 cells responded to this cytokine (Figure 1C). Consistent with the data on A375 cells, dabrafenib significantly inhibited spontaneous and VEGF-A-induced ECM invasion in SK-Mel28 cells. However, differently from what occurring in A375R cells, invasiveness of SK-Mel28R cells was not further stimulated by exposure to dabrafenib (Figure 1C).

Table 1 illustrates the amount of VEGF-A and MMP-9 determined in the culture supernatants of SK-Mel28 and SK-Mel28R cells treated for 48 h with 100 nM dabrafenib or the drug vehicle alone (i.e. dimethyl sulfoxide, DMSO). The level of the two polypeptides previously determined in A375 and A375R under the same experimental conditions [31] is also reported for comparison. Basal secretion of VEGF-A and MMP-9 was higher in SK-Mel28R cells as compared with parental SK-Mel28 cells. Moreover, dabrafenib treatment markedly impaired VEGF-A and MMP-9 secretion in SK-Mel28 cells, whereas it did not affect VEGF-A and MMP-9 secretion in SK-Mel28R cells.

**Table 1:** VEGF-A and MMP-9 secretion in A375 and SK-Mel28 cell lines and their dabrafenib-resistant counterparts

Cell Line	VEGF-A (ng/10^6^ cells)^a^			MMP-9 (pg/10^6^ cells)^a^		
	DMSO^b^	Dabrafenib^b^	*P*^c^	DMSO^b^	Dabrafenib^b^	*P*^c^
SK-Mel28	1.48 ± 0.13	0.50 ± 0.06	<0.01	5.15 ± 0.64	0.50 ± 0.07	<0.01
SK-Mel28R	76.00 ± 7.61^**^	82.98 ± 9.38^**^	NS	83.75 ± 9.91^**^	95.75 ± 16.82^**^	NS
A375 (ref. 31)	7.48 ± 1.27	3.50 ± 0.98	<0.05	24.25 ± 2.10	24.75 ± 6.52	NS
A375R (ref. 31)	15.83 ± 1.47^**^	26.04 ± 2.66^**^	<0.05	47.88 ± 3.79^**^	75.75 ± 4.09^**^	<0.01

^a^The amount of VEGF-A and MMP-9 in cell culture supernatants was determined by ELISA.

^b^cells were cultured with 100 nM dabrafenib or DMSO alone for 48 h and then culture supernatants were collected and assayed for VEGF-A and MMP-9 content.

^c^*P*, probability calculated according to Student’s *t*-test comparing dabrafenib-treated with DMSO-treated cells.

^**^P < 0.01 comparing A375R cells with A375 cells, and SK-Mel28R cells with SK-Mel28 cells.

### Effects of *PTTG1* silencing on melanoma cell proliferation, invasiveness and response to dabrafenib

To investigate whether PTTG1 plays a role in melanoma cell proliferation, invasiveness and response to dabrafenib, we first examined its expression in A375, A375R, SK-Mel28 and SK-Mel28R cell lines exposed to 100 nM dabrafenib or to DMSO for 48 h.

**Figure 2:**
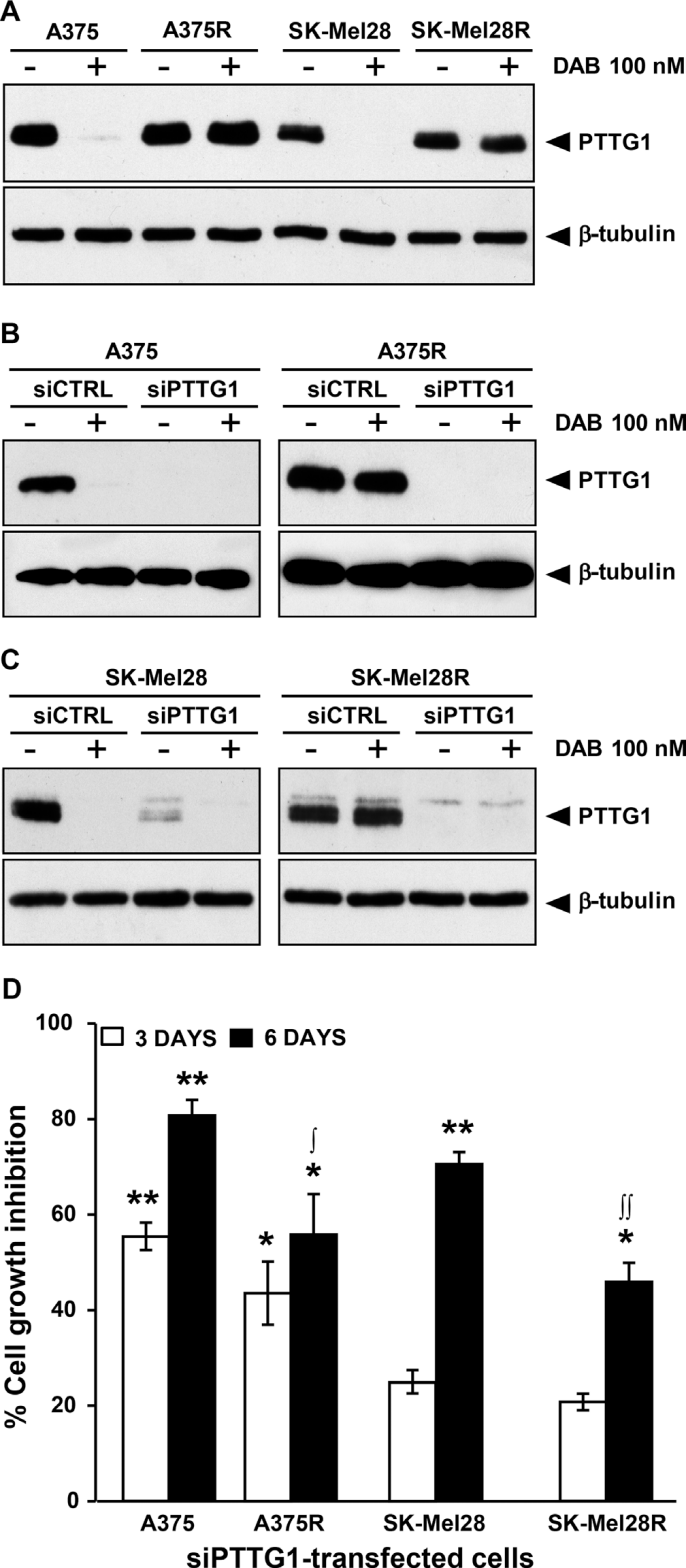
Inhibition of *PTTG1* expression impairs proliferation of melanoma cells sensitive or resistant to dabrafenib (**A**) Melanoma cells were treated with dabrafenib (DAB) or DMSO alone (–) for 48 h and then cell lysates were analyzed by immunoblotting using antibodies against PTTG1, or against β-tubulin as a loading control. The results are representative of three independent experiments. (**B**) and (**C**) Melanoma cells were transiently transfected with siPTTG1 or siCTRL and 24 h later incubated with dabrafenib (DAB) or DMSO alone (–). After 48 h of culture, cell extracts were prepared and analyzed by immunoblotting using antibodies against PTTG1 or against β-tubulin. The results are representative of three independent experiments. (**D**) Melanoma cells were transfected with siPTTG1 or siCTRL and three and six days later assessed for proliferation using the MTT assay. Data are expressed in terms of percentage of cell growth inhibition of siPTTG1-transfected cells with respect to matched siCTRL-transfected cells. Each value represents the arithmetic mean of four independent experiments. Bars, SEM. ^**^P < 0.01 and ^*^P < 0.05 siPTTG1-transfected cells *vs* matched siCTRL-transfected cells; ^**∫∫**^*P* < 0.01 and ^**∫**^*P* < 0.05 dabrafenib-resistant cells *vs* matched drug-sensitive cells.

As illustrated in Figure 2A, basal levels of PTTG1 protein were comparable between the drug-resistant cell lines and their matched parental counterparts. However, while exposure to dabrafenib completely inhibited PTTG1 expression in A375 and SK-Mel28 cells, it did not substantially affect the levels of this protein in A375R and SK-Mel28R cells.

We next evaluated whether down-modulation of PTTG1 was associated with an impairment of cell proliferation. To this end, A375, A375R, SK-Mel28 and SK-Mel28R cells were transfected with either a siRNA targeting *PTTG1* (siPTTG1) or a negative control siRNA (siCTRL) and analyzed for proliferation three and six days after transfection using the MTT assay. To confirm that PTTG1 expression was efficiently down-regulated up to the end of the proliferation assay, PTTG1 protein levels in siCTRL- and siPTTG1-transfected cells were determined by Western blot analysis six days after transfection. Evaluation of PTTG1 levels was also performed in the four cell lines that had been transfected with siCTRL or siPTTG1 and 24 h later incubated with 100 nM dabrafenib or DMSO for additional 48 h.

As illustrated in Supplementary Figure 1A, PTTG1 levels in the four cell lines remained markedly down-regulated up to six days after siPTTG1 transfection. Furthermore, expression of PTTG1 was efficiently inhibited in the siPTTG1-transfected cells either exposed to DMSO or to dabrafenib for 48 h (Figure 2B and 2C).

Three days after siPTTG1 transfection, proliferation of A375 and A375R cells was significantly inhibited, whereas that of SK-Mel28 and SK-Mel28R cells was only minimally affected (Figure 2D). On the other hand, six days after transfection, down-regulation of PTTG1 was associated with a significant impairment of proliferation in all the cell lines, even though the growth inhibitory effects of *PTTG1* silencing were higher in the dabrafenib-sensitive cell lines than in their drug-resistant counterparts (Figure 2D).

**Figure 3:**
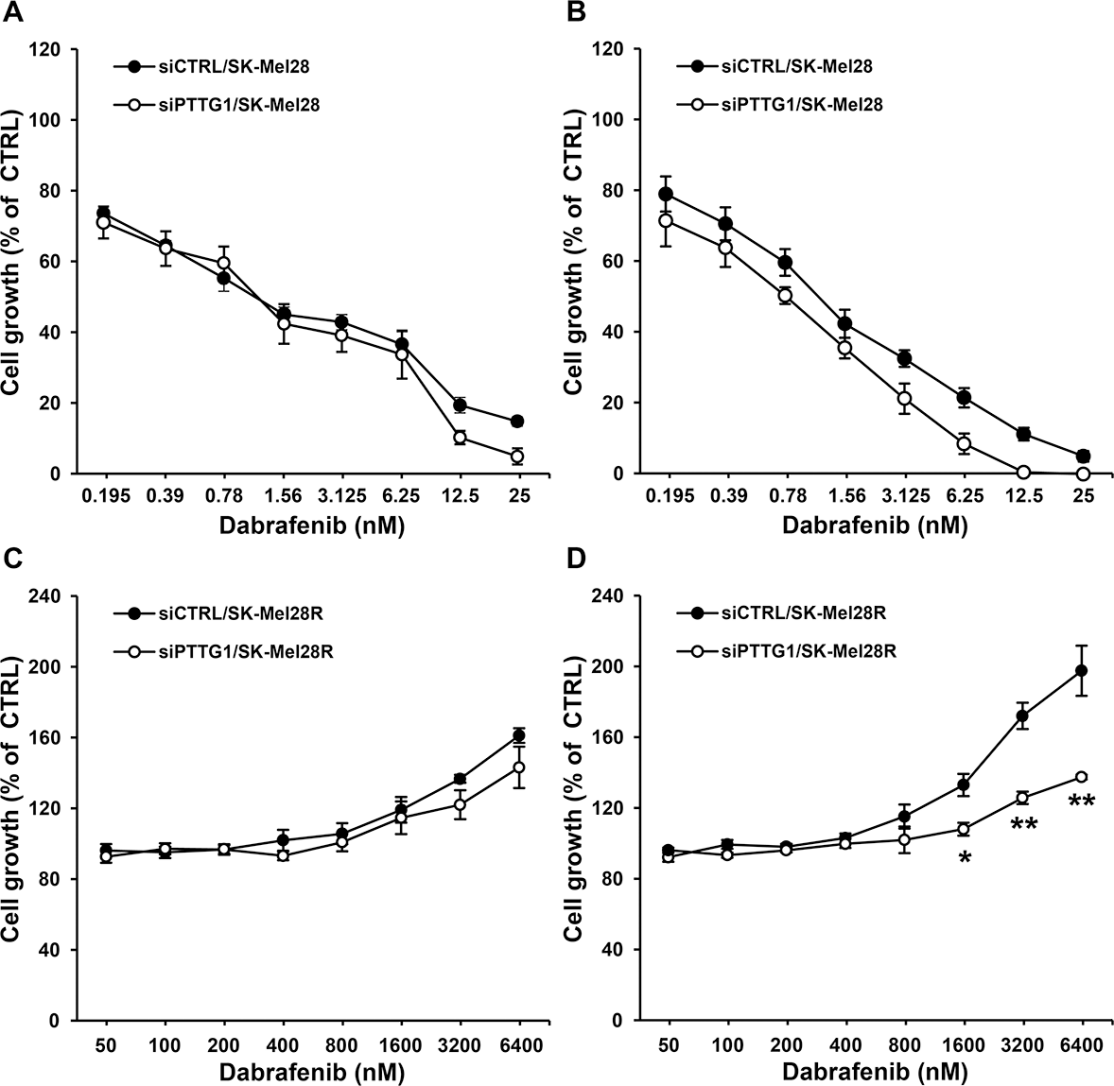
Effect of *PTTG1* silencing on SK-Mel28 and SK-Mel28R cell sensitivity to dabrafenib SK-Mel28 (**A**, **B**) and SK-Mel28R (**C**, **D**) cells were transiently transfected with siPTTG1 or siCTRL and 24 h later incubated with dabrafenib or with DMSO alone for three (A, C) or five (B, D) days. Proliferation was then assessed by the MTT assay. Data are expressed in terms of percentage of growth of cells treated with dabrafenib with respect to cells treated with DMSO alone (CTRL). Each value represents the arithmetic mean of four independent experiments. Bars, SEM. For each experiment, the dabrafenib IC_50_ value was calculated as described in the “Materials and methods” section. The IC_50_ mean value ± SEM after three days of treatment with dabrafenib was 1.38 ± 0.23 nM for siCTRL/SK-Mel28 cells and 1.08 ± 0.15 nM for siPTTG1/SK-Mel28 cells. The IC_50_ mean value ± SEM after five days of treatment with dabrafenib was 1.12 ± 0.15 nM for siCTRL/SK-Mel28 cells and 0.68 ± 0.05 nM for siPTTG1/SK-Mel28 cells (*P* < 0.05). IC_50_ values were not assessable for the SK-Mel28R cell line. ^**^P < 0.01 and ^*^P < 0.05 comparing siPTTG1/SK-Mel28R cells with siCTRL/SK-Mel28R cells at the indicated concentrations of dabrafenib.

To assess whether *PTTG1* silencing affected melanoma cell sensitivity to the growth suppressive effect of dabrafenib, A375, A375R, SK-Mel28 and SK-Mel28R cells were transfected with either siPTTG1 (hereafter referred to as siPTTG1/cells) or siCTRL (hereafter referred to as siCTRL/cells), and 24 h later incubated with graded concentrations of the drug. Proliferation was determined after three and five days of culture by the MTT assay. siPTTG1/A375 and siPTTG1/A375R cells did not show any significant increase in dabrafenib sensitivity with respect to their siCTRL-transfected counterparts at both time points analyzed (data not shown). Similarly, no effect of *PTTG1* silencing on sensitivity to dabrafenib was observed in SK-Mel 28 and SK-Mel28R cells when proliferation was evaluated after three days of drug exposure (Figure 3A and Figure 3C). On the other hand, siPTTG1/SK-Mel28 cells displayed a reduction of about 40% of the dabrafenib IC_50_ value when their proliferation was determined after five days of drug treatment (Figure 3B). Moreover, at this time point, proliferation of siPTTG1/SK-Mel28R cells was lower than that of siCTRL/SK-Mel28R cells at drug concentrations ranging between 1600 nM and 6400 nM (Figure 3D).

To determine whether PTTG1 could be involved in the regulation of the invasive capacity of dabrafenib-sensitive or dabrafenib-resistant cells and in the modulation of this cellular process by the drug, A375, A375R, SK-Mel28 and SK-Mel28R cells were transfected with siCTRL or siPTTG1 and analyzed for ECM invasion after a 48 h-exposure to 100 nM dabrafenib or DMSO.

**Figure 4:**
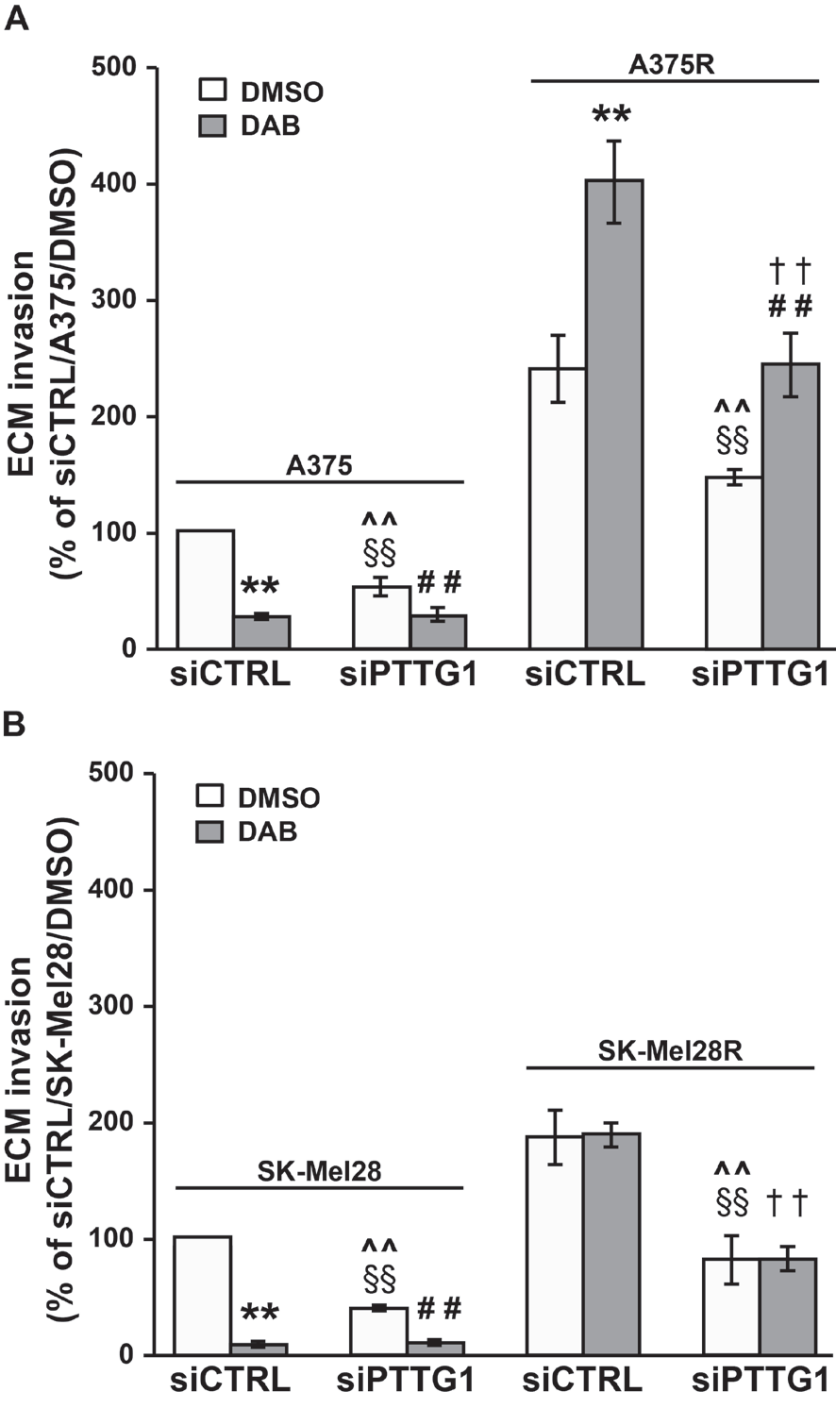
Inhibition of *PTTG1* expression impairs invasiveness of melanoma cells sensitive or resistant to dabrafenib A375 and A375R cells (**A**) as well as SK-Mel28 and SK-Mel28R cells (**B**) were transiently transfected with siPTTG1 or siCTRL and 24 h later incubated with 100 nM dabrafenib (DAB) or DMSO alone. After 48 h of culture, the cells were assayed for ECM invasion. Data are expressed in terms of percentage of invaded cells with respect to siCTRL/A375 or siCTRL/SK-Mel28 cells treated with DMSO. Each value represents the arithmetic mean ± SEM of four (A375 and A375R) or three (SK-Mel28 and SK-Mel28R) independent experiments. ^**^P < 0.01 siCTRL/DAB *vs* siCTRL/DMSO; ^##^*P* < 0.01 siPTTG1/DAB *vs* siPTTG1/DMSO; ^§§^*P* < 0.01 siPTTG1/DMSO *vs* siCTRL/DMSO; ^^^^*P* < 0.01 siPTTG1/DMSO *vs* siCTRL/DAB; ^††^*P* < 0.01 siPTTG1/DAB *vs* siCTRL/DAB.

We found that *PTTG1* silencing alone caused a significant inhibition of the invasive capacity of the four cell lines (Figure 4A and 4B). Dabrafenib treatment markedly impaired ECM invasion in A375 and SK-Mel28 cells transfected with either siCTRL or siPTTG1 (Figure 4A and 4B). In both cell lines, inhibition of invasiveness induced by dabrafenib alone was higher than that caused by *PTTG1* silencing alone and similar to that induced by dabrafenib plus siPTTG1 (Figure 4A and 4B).

In both siCTRL/A375R and siPTTG1/A375R cells, exposure to dabrafenib promoted ECM invasion. However, invasiveness of drug-treated siPTTG1/A375R cells was significantly lower than that of drug-treated siCTRL/A375R cells and comparable to that of siCTRL/A375R cells exposed to DMSO (Figure 4A). As expected, exposure to dabrafenib did not modify the invasive capacity of siCTRL/SK-Mel28R cells. The drug was ineffective also in the presence of *PTTG1* silencing (Figure 4B).

### Effects of *PTTG1* over-expression on melanoma cell proliferation, invasiveness and response to dabrafenib

To evaluate the effect of *PTTG1* over-expression on melanoma cell proliferation, A375 cells were transiently transfected with an expression vector encoding a FLAG-tagged PTTG1 protein (CMV-PTTG1) or with the empty vector (CMV-EV). Six days after transfection, cells were analyzed for proliferation using the MTT assay and processed for Western blot analysis to confirm PTTG1-FLAG expression. Western blot analysis of PTTG1 levels was also performed in the cells that had been transfected with CMV-PTTG1 or CMV-EV and 24 h later incubated with 100 nM dabrafenib or DMSO for additional 48 h.

**Figure 5:**
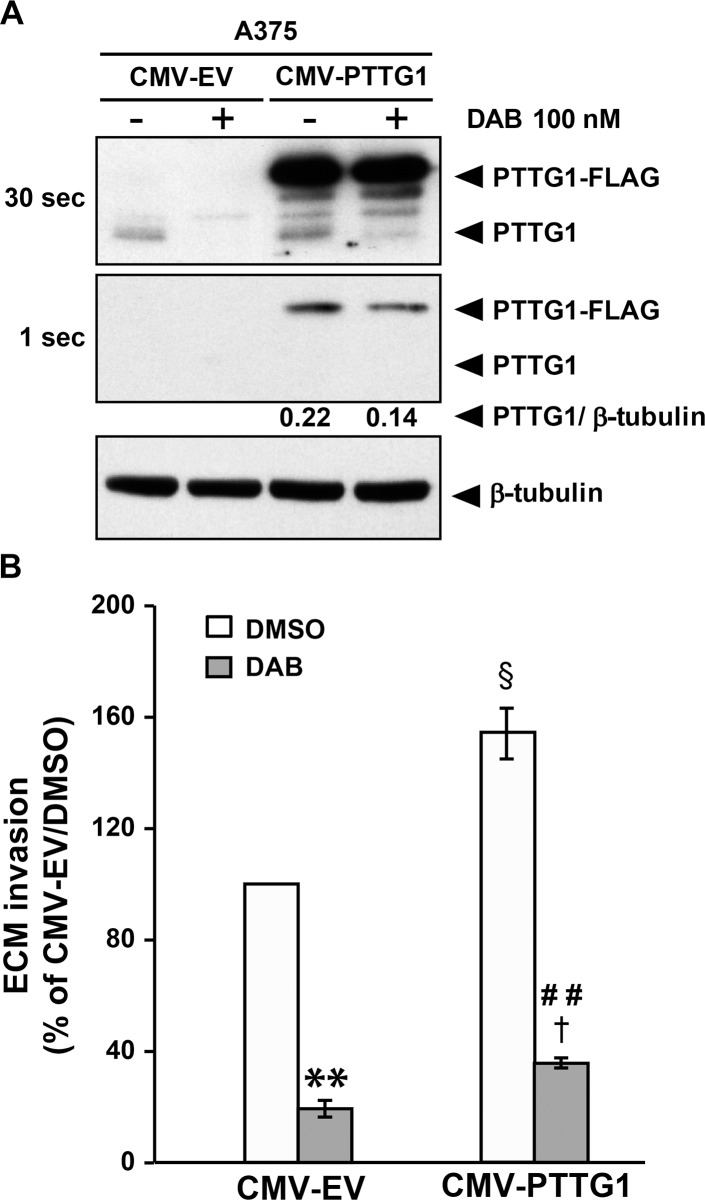
*PTTG1* over-expression increases invasiveness of melanoma cells (**A**) A375 cells were transiently transfected with CMV-PTTG1 or CMV-EV and 24 h later exposed to 100 nM dabrafenib (DAB) or DMSO alone (–) for 48 h. Cell lysates were then analyzed by immunoblotting using antibodies against PTTG1 or against β-tubulin. Two different exposure (30 sec and 1 sec) of the same membrane are shown to evidence dabrafenib-induced decrease of both endogenous PTTG1 (PTTG1) and transfected (PTTG1-FLAG) protein in CMV-PTTG1-transfected cells. The ratio between the densitometric level of PTTG1-FLAG and that of β-tubulin is shown for the 1 sec-exposure. The results are representative of three independent experiments. (**B**) A375 cells were transiently transfected with CMV-PTTG1 or CMV-EV. Twenty-four hours later the cells were incubated with 100 nM dabrafenib (DAB) or DMSO alone and assayed for ECM invasion after 48 h of culture. Data are expressed in terms of percentage of invaded cells with respect to CMV-EV/A375 cells treated with DMSO. Each value represents the arithmetic mean ± SEM of three independent experiments. ^**^P < 0.01 CMV-EV/DAB *vs* CMV-EV/DMSO; ^##^*P* < 0.01 CMV-PTTG1/DAB *vs* CMV-PTTG1/DMSO; ^§^*P* < 0.05 CMV-PTTG1/DMSO *vs* CMV-EV/DMSO; ^†^*P* < 0.05 CMV-PTTG1/DAB *vs* CMV-EV/DAB.

As illustrated in Figure 5A, FLAG-tagged PTTG1 protein was successfully over-expressed in CMV-PTTG1-transfected cells, and remained at elevated levels up to six days after transfection (Supplementary Figure 1B). Exposure to dabrafenib for 48 h abrogated PTTG1 expression in CMV-EV cells and reduced the amount of endogenous and FLAG-tagged PTTG1 protein in CMV-PTTG1 cells.

*PTTG1* over-expression was not associated with an increase of A375 cell proliferation (data not shown). Moreover, CMV-PTTG1 cells did not show increased resistance to the antiproliferative effects of dabrafenib as determined by MTT assays (data not shown).

A375 cells transfected with either the CMV-PTTG1 or CMV-EV vector were also analyzed for the ability to invade the ECM after a 48 h-exposure to 100 nM dabrafenib or DMSO.

The results illustrated in Figure 5B show that invasiveness of CMV-PTTG1 cells was significantly higher than that of the cells transfected with the empty vector. In both control and PTTG1 over-expressing cells, dabrafenib treatment significantly inhibited the ability to invade the ECM. However, invasiveness of dabrafenib-treated CMV-PTTG1/A375 cells was higher than that of drug-treated CMV-EV/A375 cells, in agreement with the finding that former cells expressed PTTG1 levels higher than those of CMV-EV cells.

### LEE011 impairs PTTG1 expression, proliferation and invasiveness of A375R cells

We previously demonstrated that the CDK inhibitor PHA848125 - shown to possess a safety profile and a promising activity against thymic carcinoma in a phase I clinical study [32] - inhibited proliferation of A375 cells, at least in part, through down-regulation of PTTG1 [30]. We, therefore, sought to investigate whether LEE011 (ribociclib), a CDK4/6 inhibitor approved by FDA for breast cancer treatment and under clinical investigation in various type of tumors, including melanoma (www.clinicaltrial.gov), was able to impair PTTG1 expression in A375R cells and whether this molecular event was associated with inhibition of proliferation and invasion and/or modulation of response to dabrafenib.

A375R cells were exposed to DMSO, LEE011 (4 μM or 16 μM), dabrafenib (100 nM) or a combination of LEE011 (4 μM or 16 μM) plus dabrafenib and analyzed for PTTG1 expression, proliferation and ECM invasion 48 h after drug treatment.

**Figure 6:**
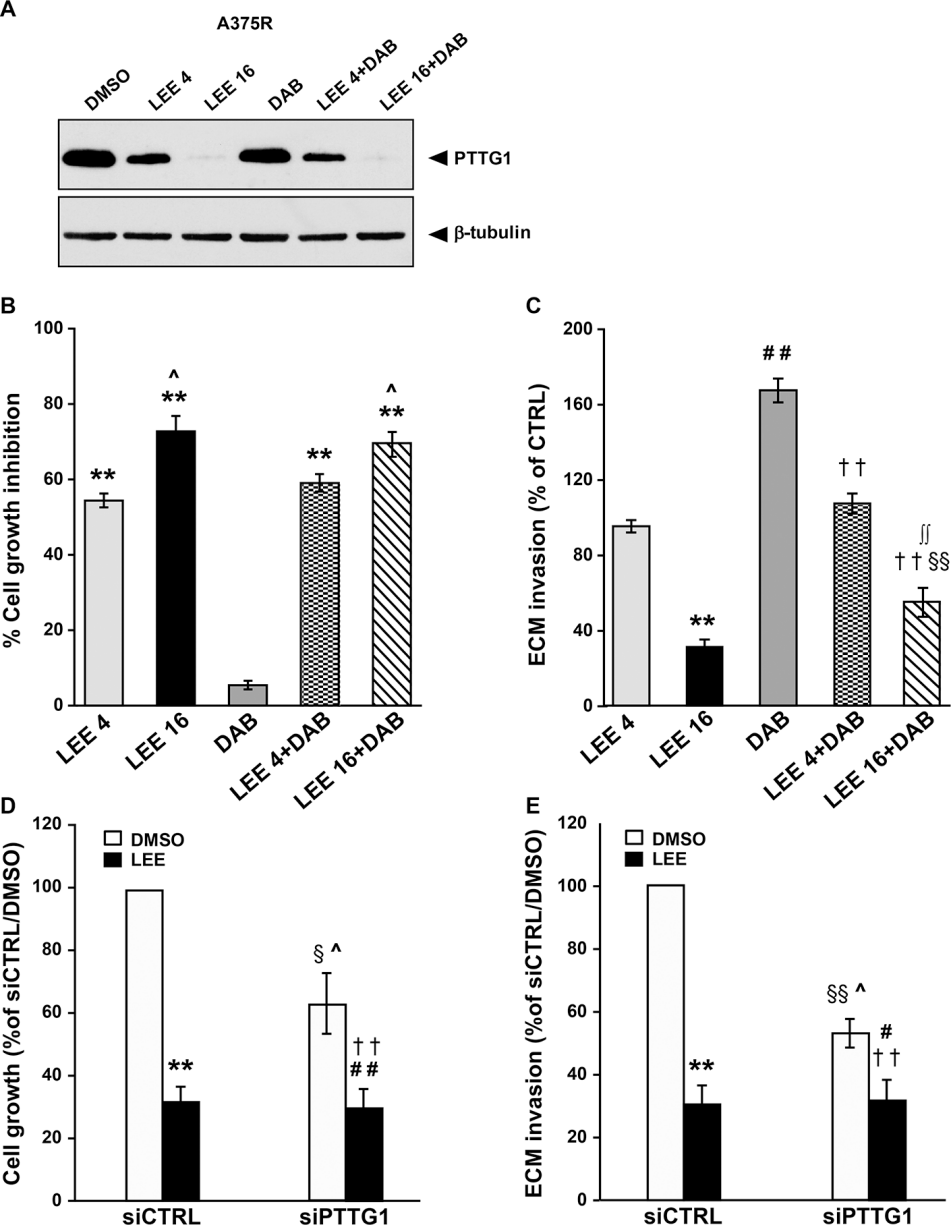
LEE011 reduces PTTG1 expression, invasiveness and proliferation in A375R cells (**A**) A375R cells were incubated with DMSO alone, 4 µM LEE011 (LEE 4), 16 µM LEE011 (LEE 16), 100 nM dabrafenib (DAB) or a combination of dabrafenib plus 4 µM or 16 µM LEE011 for 48 h and then cell lysates were analyzed by immunoblotting using antibodies against PTTG1 or against β-tubulin. The results are representative of three independent experiments. (**B**) A375R cells were treated as described in (A) and then recovered and counted. Data are expressed in terms of percentage of cell growth inhibition of drug-treated cells with respect to cells treated with DMSO alone. Each value represents the arithmetic mean of four independent experiments. Bars, SEM. ^**^P < 0.01 drug-treated cells *vs* DMSO-treated cells. ^^^*P* < 0.05 16 µM LEE011 *vs* 4 µM LEE011 and DAB+16 µM LEE011 *vs* DAB+4 µM LEE011. (**C**) A375R cells were treated as described in (A) and then assayed for ECM invasion. Data are expressed in terms of percentage of invaded cells with respect to A375R cells treated with DMSO (CTRL). Each value represents the arithmetic mean ± SEM of four independent experiments. ^**^P < 0.01 LEE 16 *vs* DMSO; ^##^*P* < 0.01 DAB *vs* DMSO; ^§§^*P* < 0.01 LEE 16+DAB *vs* DMSO; ^††^*P* < 0.01 LEE 4+DAB and LEE 16+DAB *vs* DAB; ^**∫∫**^*P* < 0.01 LEE 16+DAB *vs* LEE 16. (**D**) A375R cells were transiently transfected with siPTTG1 or siCTRL and 24 h later incubated with DMSO alone or 16 µM LEE011 (LEE). After 48 h of culture, the cells were recovered and counted. Data are expressed in terms of percentage of growth with respect to siCTRL/A375R cells treated with DMSO. Each value represents the arithmetic mean ± SEM of three independent experiments. ^**^P < 0.01 siCTRL/LEE *vs* siCTRL/DMSO; ^##^*P* < 0.01 siPTTG1/LEE *vs* siPTTG1/DMSO; ^§^*P* < 0.05 siPTTG1/DMSO *vs* siCTRL/DMSO; ^^^*P* < 0.05 siPTTG1/DMSO *vs* siCTRL/LEE; ^††^*P* < 0.01 siPTTG1/LEE *vs* siCTRL/DMSO. (**E**) A375R cells were transfected and treated and indicated in (D). After 48 h of culture, the cells were assayed for ECM invasion. Data are expressed in terms of percentage of invaded cells with respect to siCTRL/A375R cells treated with DMSO. Each value represents the arithmetic mean ± SEM of three independent experiments. ^**^P < 0.01 siCTRL/LEE *vs* siCTRL/DMSO; ^#^*P* < 0.05 siPTTG1/LEE *vs* siPTTG1/DMSO; ^§§^*P* < 0.01 siPTTG1/DMSO *vs* siCTRL/DMSO; ^^^*P* < 0.05 siPTTG1/DMSO *vs* siCTRL/LEE; ^††^*P* < 0.01 siPTTG1/LEE *vs* siCTRL/DMSO.

As illustrated in Figure 6 A, PTTG1 expression was reduced in A375R cells treated with 4 μM LEE011 and almost abrogated in the cells exposed to 16 μM of the drug, either alone or in combination with dabrafenib.

As expected, dabrafenib did not affect proliferation of A375R cells, whereas a concentration-dependent inhibition of cell growth occurred in LEE011-treated cells (Figure 6B). Moreover, the antiproliferative effect of the combinations of LEE011 plus dabrafenib was comparable to that exerted by the corresponding concentrations of LEE011 alone (Figure 6B).

Treatment with 16 μM LEE011 significantly impaired the invasive capacity of A375R cells (Figure 6C). A375R cells exposed to dabrafenib in combination with this concentration of LEE011 displayed higher ECM invasion as compared with the cells treated with the CDK inhibitor alone. However, their invasive capacity remained significantly lower than that of the cells exposed to DMSO or to dabrafenib alone (Figure 6C). The lowest concentration of LEE011 failed to reduce basal invasiveness of A375R cells. However, it abrogated up-regulation of ECM invasion induced by dabrafenib in this cell line (Figure 6C)**.**

To confirm that the effects of LEE011 on A375R cell proliferation and invasiveness were due, at least in part, to PTTG1 inhibition, the cells were transfected with siCTRL or siPTTG1 and assayed for proliferation and ECM invasion after a 48 h-exposure to DMSO, or 16 μM LEE011.

As expected, proliferation of A375R cells was reduced of about 70% and of about 40% by LEE011 treatment and *PTTG1* silencing, respectively (Figure 6D). Moreover, the combination of siPTTG1 and LEE011 caused an impairment of cell growth comparable to that produced by LEE011 alone. Consistent with these results, the inhibitory effect of LEE011 on A375R invasiveness was more pronounced than that of siPTTG1 and not enhanced in the cells that had been knocked-down for *PTTG1* (Figure 6E).

### Down-regulation of MMP-9 secretion is involved in siPTTG1-induced inhibition of A375R cell invasiveness

It has been previously shown that PTTG1 can promote tumor cell invasiveness by increasing the expression of several effector molecules, including MMP-9 [21, 33, 34] which has been associated with an invasive phenotype in melanoma [35, 36]. We therefore decided to investigate whether inhibition of MMP-9 secretion could underlie, at least in part, the inhibitory effects exerted by *PTTG1* silencing on the invasive capacity of dabrafenib-resistant cells. To this end, A375R cells were transfected with siCTRL or siPTTG1, and assayed for MMP-9 release after a 48 h-exposure to 100 nM dabrafenib or DMSO.

**Figure 7:**
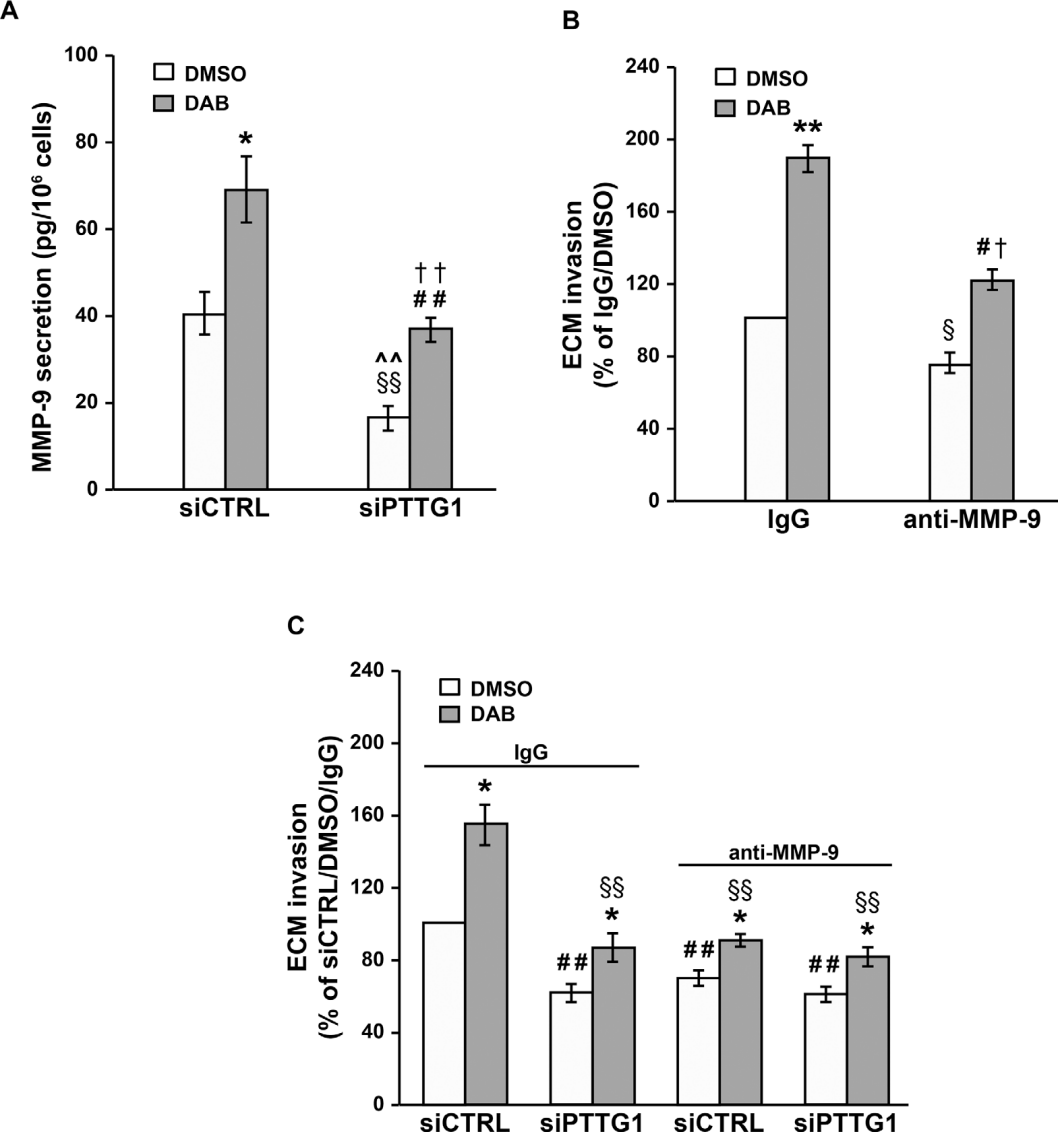
Inhibition of MMP-9 secretion contributes to the impairment of A375R cell invasiveness induced by *PTTG1* silencing **(A)** A375R cells were transiently transfected with siPTTG1 or siCTRL and 24 h later incubated with 100 nM dabrafenib (DAB) or DMSO alone. After 48 h of drug exposure, the amount of MMP-9 in the culture supernatants was determined by ELISA. Each value represents the arithmetic mean ± SEM of six independent experiments. ^*^P < 0.05 siCTRL/DAB *vs* siCTRL/DMSO; ^##^*P* < 0.01 siPTTG1/DAB *vs* siPTTG1/DMSO; ^§§^*P* < 0.01 siPTTG1/DMSO *vs* siCTRL/DMSO; ^^^^*P* < 0.01 siPTTG1/DMSO *vs* siCTRL/DAB; ^††^*P* < 0.01 siPTTG1/DAB *vs* siCTRL/DAB. (**B**) A375R cells were treated with 100 nM dabrafenib (DAB) or DMSO alone for 48 h and then assayed for their ability to invade the ECM in the presence of an anti-MMP-9 mAb or a control antibody (IgG). Data are expressed in terms of percentage of invaded cells with respect to A375R cells treated with DMSO and assayed in the presence of the control antibody. Each value represents the arithmetic mean ± SEM of three independent experiments. ^**^P < 0.01 DAB/IgG vs DMSO/IgG; ^#^*P* < 0.05 DAB/anti-MMP-9 vs DMSO/anti-MMP-9; ^§^*P* < 0.05 DMSO/anti-MMP-9 vs DMSO/IgG; ^†^*P* < 0.05 DAB/anti-MMP-9 vs DAB/IgG. (**C**) A375R cells were transiently transfected with siPTTG1 or siCTRL and 24 h later incubated with 100 nM dabrafenib (DAB) or DMSO alone. After 48 h of drug exposure, the cells were assayed for their ability to invade the ECM in the presence of an anti-MMP-9 mAb or a control antibody (IgG). Each value represents the arithmetic mean ± SEM of three independent experiments. ^*^P < 0.05 dabrafenib-treated cells *vs* matched DMSO-treated cells; ^##^*P* < 0.01 siPTTG1/IgG/DMSO, siCTRL/MMP-9/DMSO, siPTTG1/MMP-9/DMSO vs siCTRL/IgG/DMSO; ^§§^*P* < 0.01 siPTTG1/IgG/DAB, siCTRL/MMP-9/DAB, siPTTG1/MMP-9/DAB vs siCTRL/IgG/DAB.

As illustrated in Figure 7A, inhibition of PTTG1 expression alone caused a marked reduction in the amount of MMP-9 released by the cells. In both siCTRL/A375R and siPTTG1/A375R cells, exposure to dabrafenib increased MMP-9 secretion. However, the amount of MMP-9 released by drug-treated siPTTG1/A375R cells was significantly lower than that of drug-treated siCTRL/A375R cells and comparable to that of siCTRL/A375R cells exposed to DMSO.

A375R cells were then incubated with 100 nM dabrafenib or DMSO and after 48 h of culture assayed for ECM invasion in the presence of an anti-MMP-9 mAb, or of a control antibody. Both DMSO-treated and dabrafenib-treated cells displayed a significant inhibition of ECM invasion when assayed in the presence of the anti-MMP-9 mAb (Figure 7B).

We next evaluated the effect of the anti-MMP-9 mAb on the invasive capacity of siCTRL/A375R and siPTTG1/A375R cells exposed to 100 nM dabrafenib or DMSO for 48 h.

With reference to the cells treated with DMSO alone, the results presented in Figure 7C show that the invasive capacity of siPTTG1/A375R cells assayed in the presence of the control antibody was comparable to that of siCTRL/A375R cells tested in the presence of the anti-MMP-9 mAb. Moreover, no further inhibition of ECM invasion occurred when siPTTG1/A375R cells were assayed in the presence of the anti-MMP-9 mAb (Figure 7C). These findings support the hypothesis that *PTTG1* silencing impairs invasiveness of A375R cells through a down-regulation of MMP-9 levels.

In all instances, the invasive capacity of dabrafenib-treated cells was higher than that of the corresponding DMSO-treated counterparts (Figure 7C). Consistent with the results obtained with the cells exposed to DMSO alone, dabrafenib-treated cells subjected to either *PTTG1* silencing or to MMP-9 neutralization or to both conditions displayed a comparable level of ECM invasion. This was significantly reduced with respect to that of dabrafenib-treated siCTRL/A375R cells and comparable to that of DMSO-treated siCTRL/A375R cells, both assayed in the presence of the control antibody (Figure 7C).

### Evaluation of PTTG1 plasma levels in melanoma patients subjected to therapy with dabrafenib, vemurafenib or dabrafenib plus trametinib

Previous studies by Wang *et al.* [37] demonstrated that the PTTG1 protein could be detected in peripheral blood from non-cancer patients and to a significantly higher extent in peripheral blood from patients with colorectal neuroendocrine tumor. We therefore carried out a preliminary investigation to evaluate whether the PTTG1 protein was present also in plasma of melanoma patients and whether changes in PTTG1 levels, possibly related to clinical response, could occur during therapy with BRAFi (dabrafenib or vemurafenib) or the combination of dabrafenib plus trametinib. The study was conducted on a total of 22 patients from whom plasma samples collected before the start of the therapy (T0), after two months of treatment (T2) and at disease progression (TP) were already available.

Among the 22 patients, 11 were subjected to dabrafenib or vemurafenib monotherapy, whereas 3 were treated with dabrafenib alone for 8, 5 or 4 months and then with dabrafenib plus trametinib. The remaining patients were subjected to the combination of dabrafenib plus trametinib (Table 2). Five patients experienced disease progression within 2 months from the start of therapy, whereas 1 patient had stable disease for 5 months and then underwent progression (Table 2). These patients were included in the group of “non-responders” and only T0 and T2 plasma samples were considered for the analysis. The remaining patients experienced partial response as evaluated by computer tomography after 3 months of therapy (Table 2), and constituted the group of “responders”. Among the 60 plasma samples tested, 18 displayed PTTG1 levels under the lower limit of ELISA detection (T0, *n =* 7; T2, *n =* 8; TP, *n =* 3).

**Table 2:** Demographics and clinical characteristics of melanoma patients from whom plasma was collected

Patient case	Sex	Age (years)	Stage^a^	Previous therapy	Targeted therapy^b^	Response^c^	TTF (days)^d^
1	F	45	M1c	None	DAB (8) → COMBO	PR	1324
2	F	48	M1c	Fotemustine	DAB	PR	182
3	M	38	M1c	None	DAB	PR	144
4	F	64	M1b	None	COMBO	PR	234
5	M	71	M1c	None	DAB (5) → COMBO	PR	1275
6	M	43	M1c	None	DAB	SD	-
7	M	47	M1c	Fotemustine	DAB	PR	147
8	M	66	M1a	None	DAB (4) → COMBO	CR	488
9	F	82	M1c	Dacarbazine	VEMU	PD	-
10	M	81	M1c	None	VEMU	PD	-
11	M	57	M1c	None	VEMU	PR	223
12	M	60	M1b	None	COMBO	PR	1082
13	M	45	M1c	None	COMBO	PR	188
14	M	70	M1c	None	COMBO	PR	117
15	M	65	M1c	None	VEMU	PD	-
16	M	49	M1c	None	VEMU	PR	147
17	M	39	M1b	None	DAB	PD	-
18	M	81	M1c	None	VEMU	PD	-
19	F	46	M1c	None	COMBO	PR	244
20	M	56	M1c	Nivolumab	COMBO	PR	504
21	M	57	M1c	None	COMBO	PR	172
22	M	35	M1c	None	COMBO	PR	180

^a^Stage at first plasma collection (i.e. T0).

^b^DAB, dabrafenib. In parenthesis, months of monotherapy before trametinib addiction. COMBO, dabrafenib + trametinib. VEMU, vemurafenib.

^c^Clinical response as evaluated three months after therapy commencement. PR, partial response; SD, stable disease; PD, progressive disease.

^d^TTF, time to treatment failure.

**Figure 8:**
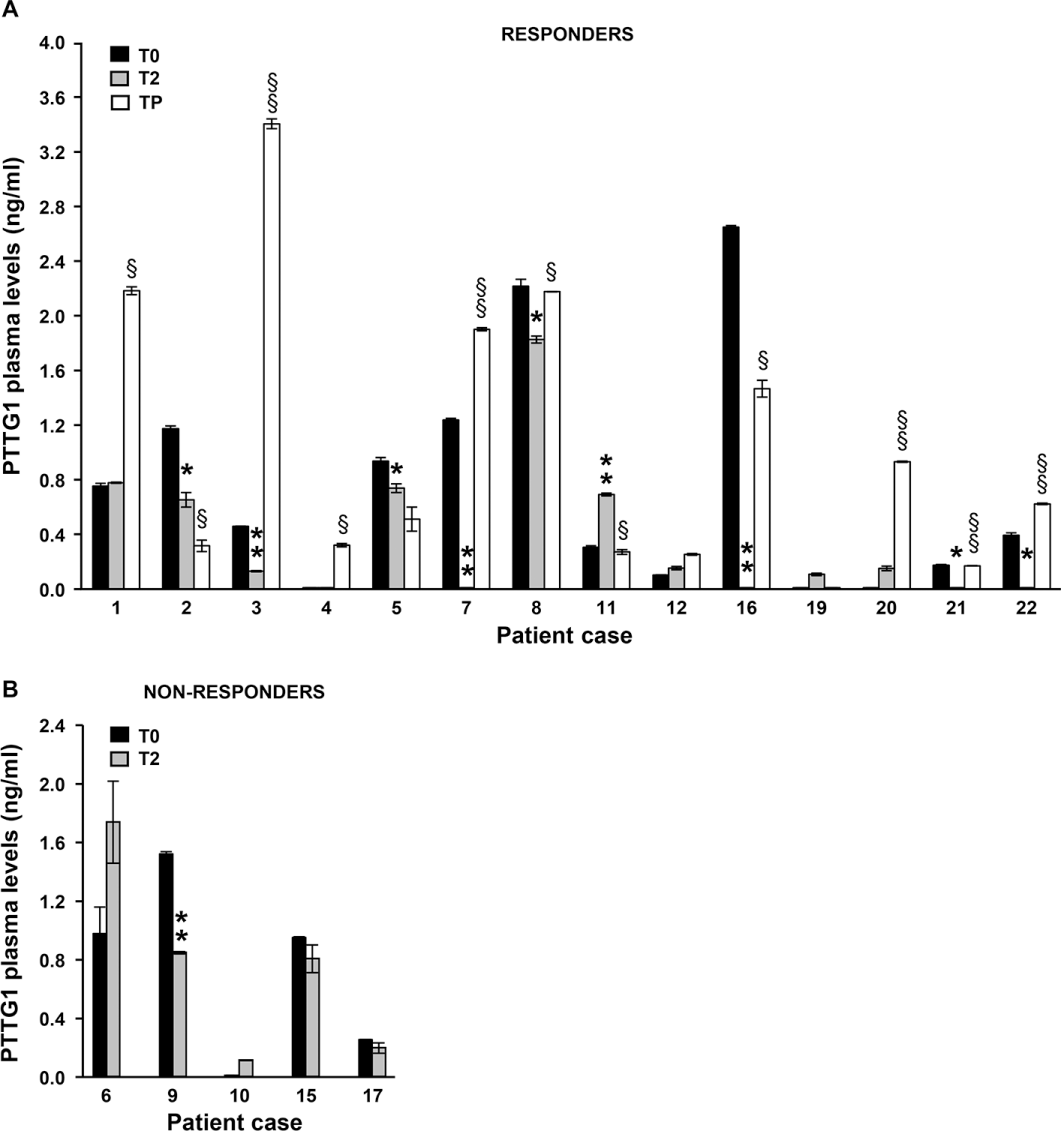
Quantification of PTTG1 protein in plasma of melanoma patients treated with BRAFi or the combination of dabrafenib plus trametinib PTTG1 levels were determined by ELISA in plasma samples of 16 responder (**A**) and 6 non-responder (**B**) melanoma patients before the start of therapy (T0), after two months of treatment (T2) and at disease progression (TP). Two patients among responders (case #13 and case #14) and 1 patient among non-responder (case #18) displayed PTTG1 plasma levels below the detection limit of the assay at all the time points analyzed and were not included in the figures. Each value represents the arithmetic mean ± SEM of two independent determinations. ^**^P < 0.01 and ^*^P < 0.05 T2 *vs* T0; ^§§^*P* < 0.01 and ^§^*P* < 0.05 TP *vs* T2.

Among the responder patients, 2 (case #13 and case #14) displayed PTTG1 plasma levels below the limit of assay detection at all the three time points analyzed, whereas the remaining patients had at least one time point with measurable PTTG1 level. The amount of PTTG1 in plasma of these patients at the different time points analyzed is illustrated in Figure 8A. A decrease of circulating PTTG1 was observed at T2 in 8 out of 11 (73%) responder patients with detectable T0-PTTG1 levels. In 6 of these patients, PTTG1 plasma levels rose again at TP. An increase in circulating PTTG1 at TP as compared with T2 was also observed in 2 patients with no measurable PTTG1 at T0 and in 1 patient with comparable PTTG1 levels at T0 and T2.

Among the non-responder patients, 1 (case #18) had both T0 and T2 levels under the limit of the assay, 1 displayed reduced PTTG1 levels at T2, while the remaining patients (67%) did not show significant changes in plasma PTTG1 levels between the two time points investigated (Figure 8B).

**Figure 9:**
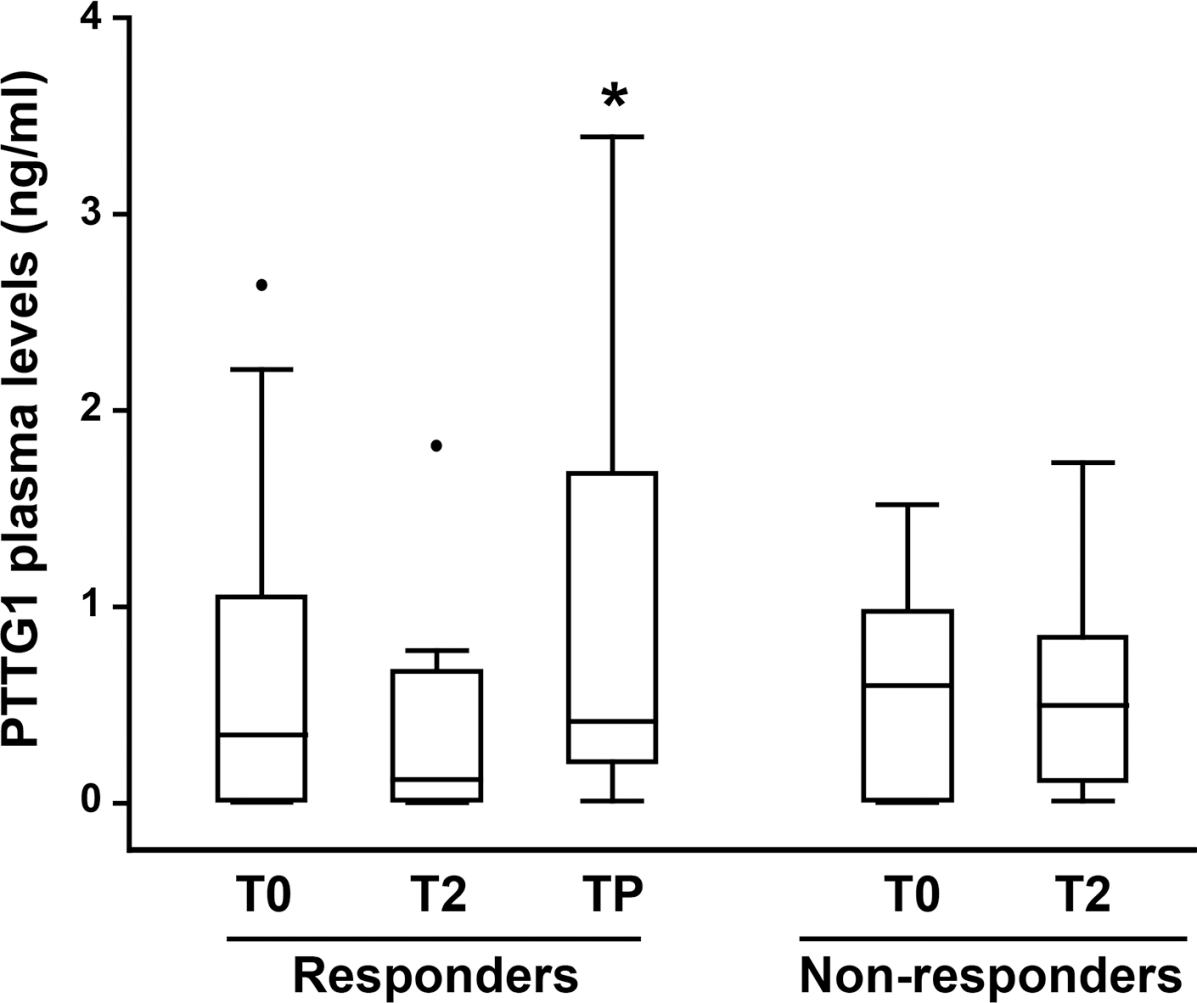
Box-and-whisker diagrams of PTTG1 plasma levels in melanoma patients treated with BRAFi or the combination of dabrafenib plus trametinib PTTG1 plasma levels were measured in 22 melanoma patients (16 responders and 6 non-responders) before therapy commencement (T0), after two months of treatment (T2) and at disease progression (TP). The edges of each box represent the 75th and 25th percentile, respectively, and whiskers the maxima and minima. The horizontal bar within each box indicates the median. The outliers are denoted by dots. Data were analysed by nonparametric Wilcoxon matched-pairs signed-rank test; ^*^P < 0.05 TP *vs* T2.

Figure 9 shows the results of statistical analyses performed on PTTG1 plasma levels detected at the different time points in all responder and non-responder patients. At baseline (T0), no significant differences were observed in PTTG1 levels between responder and non-responder group. The median PTTG1 levels at baseline was 0.35 ng/mL (IQR *=* 0.01, 1.05) for the responders group and 0.60 ng/mL (IQR *=* 0.01, 0.98) for non-responders (*P =* 0.89). Compared to T0, a considerable decrease in PTTG1 level was observed at T2 in responders (median 0.12 ng/mL; IQR *=* 0.01, 0.67) even though the T0–T2 comparison did not reach the statistical significance (*P =* 0.11). A statistically significant increase of PTTG1 levels was observed at TP in comparison with T2, with a median level of 0.42 ng/mL (IQR *=* 0.21, 1.68) (*P* < 0.05). In non-responder patients, PTTG1 levels at T2 were similar to baseline levels, with a median level of 0.50 ng/mL (IQR = 0.01, 0.85) (*P =* 0.83).

## DISCUSSION

Cutaneous melanoma is an aggressive cancer that causes the greatest number of skin cancer-related deaths worldwide. While early-stage melanoma can be cured successfully by surgical resection, patients with metastatic melanoma have a poor prognosis, with a median survival rate of less than 1 year and a 5-year survival rate of less than 5% [38]. Monotherapy with BRAFi and combined therapy with BRAFi and MEKi possess remarkable clinical activity in patients with metastatic BRAF-mutant melanoma - yielding objective response rates of 50–60% and of 65–75%, respectively - and significantly prolong progression-free and overall survival [39–41]. However, drug resistance eventually, and often rapidly, emerges, limiting the long-term efficacy of the targeted therapy [42–44]. In addition, most responses are partial and about 10–20% of patients show primary resistance. Novel therapeutic approaches able to improve response to BRAFi and MEKi and to mitigate or overcome acquired resistance, as well as biomarkers to predict and/or monitor response to therapy, are therefore urgently needed.

In this study we investigated the involvement of PTTG1 in the regulation of proliferation and invasiveness of melanoma cells sensitive or resistant to dabrafenib and whether targeting PTTG1 could affect the response of those cells to the BRAFi. We also performed a preliminary study to explore the potential value of PTTG1 plasma level to monitor patient response to treatment with BRAFi, alone or in combination with MEKi.

### Dabrafenib-sensitive cells

Our results show that in the dabrafenib-sensitive cell lines A375 and SK-Mel28, *PTTG1* silencing markedly inhibited ECM invasion. Moreover, proliferation of both cell lines was strongly reduced six days after siPTTG1 transfection. A significant inhibition of cell growth was evident in A375 but not SK-Mel28 cells after three days of *PTTG1* silencing, most probably because of the higher proliferative rate of A375 cells as compared with SK-Mel28 cells. Notably, over-expression of *PTTG1* in A375 cells promoted invasiveness but not proliferation, suggesting that the basal level of PTTG1, which in those cells is markedly up-regulated as compared with normal melanocytes [30], is already sufficient to maximally support cell growth.

In the drug-sensitive cell lines, dabrafenib treatment abrogated PTTG1 expression and this molecular event appears to contribute to the suppressive effects exerted by the drug on cell ability to invade the ECM. Indeed, as stated above, *PTTG1* silencing alone was able to impair invasiveness of A375 and SK-Mel28 cells. Moreover, in both cell lines, dabrafenib-induced inhibition of ECM invasion was not affected by *PTTG1* silencing, as expected being the drug able to suppress PTTG1 expression by itself. Noteworthy, the impairment of ECM invasion caused by *PTTG1* knock-down was lower than that produced by dabrafenib, indicating that beside PTTG1 expression, additional pathways involved in melanoma cell invasiveness are inhibited by this drug. Accordingly, the invasive capacity of A375 cells over-expressing PTTG1 was still markedly reduced by dabrafenib, even though it was significantly higher than that displayed by dabrafenib-treated control cells.

The influence of PTTG1 expression level on the growth suppressive effects of dabrafenib appears to be more dependent on the cell context, since in A375 cells neither *PTTG1* silencing nor *PTTG1* over-expression altered sensitivity to dabrafenib, whereas *PTTG1* knock-down increased drug sensitivity of SK-Mel28 cells, as evidenced by MTT assays performed after five days of exposure to dabrafenib. The molecular mechanisms underlying this different behavior of A375 and SK-Mel28 cells remain, however, to be ascertained. In agreement with the finding that several antitumor agents negatively modulate PTTG1 expression through p53 activation [24, 45], we previously demonstrated that the CDK inhibitor PHA-848125 markedly impaired PTTG1 expression and proliferation in melanoma cells endowed with wild-type p53, whereas in p53-mutated cells the drug did not substantially affect the levels of this protein and inhibited proliferation to a lesser extent [30]. Accordingly, *PTTG1* silencing increased PHA-848125 sensitivity only in p53-mutated cells, being these cells unable to undergo a p53-mediated reduction of PTTG1 upon exposure to PHA-848125 alone [30]. A375 cells are p53 wild-type, whereas SK-Mel28 cells express a mutant form of p53 [46]. However, in both A375 and SK-Mel28 cells dabrafenib equally impaired PTTG1 expression. Therefore, the different p53 status of A375 and SK-Mel28 cells does not explain why siPTTG1 increased dabrafenib sensitivity only in SK-Mel28 cells. On the other hand, PTTG1 interact with numerous proteins and, as a transcription factor, it can directly or indirectly regulate the expression of hundreds of genes involved in cell cycle, apoptosis, metabolism and signal transduction [1–4]. It is possible to speculate that differences in the expression/mutational status of PTTG1 targets between A375 and SK-Mel28 cells could be responsible for their different response to dabrafenib after siPTTG1 transfection.

Previous investigations performed in cellular models different from melanoma, point out that PTTG1 expression can be regulated by multiple mechanisms. Actually, transcription of the *PTTG1* gene was found to be directly activated by the transcription factors SP1, NF-Y, Oct-1 and T-cell factor 4, and, as stated above, suppressed by p53 [1, 4]. Positive regulation of PTTG1 expression was also demonstrated for several growth factors, including epidermal growth factor, hepatocyte growth factor, fibroblast growth factor-2, insulin-like growth factor-1, which are known to activate mitogen-activated protein kinase (MAPK) and phosphatidylinositol 3-kinase signaling pathways [1, 2, 4]. More recently, several microRNAs have also emerged as negative regulators of *PTTG1* expression [8, 11, 13, 19, 47, 48]. Interestingly, Hernandez *et al.* [49] demonstrated that dicumarol down-regulated PTTG1 expression in HCT116 colon cancer cells at least in part through inhibition of Hsp90 and the consequent impairment of the RAS/RAF/MEK/ERK signaling pathway. Although further studies are required to identify the mechanisms underlying dabrafenib-induced inhibition of PTTG1 expression, it is reasonable to hypothesize that drug-induced impairment of the BRAF/MEK/ERK signaling cascade contributes to this molecular event.

### Dabrafenib-resistant cells

In this study we analyzed two cell lines with acquired resistance to dabrafenib, namely A375R and SK-Mel28R. With respect to their matched drug-sensitive counterpart, both cell lines displayed increased invasiveness and secreted higher amount of VEGF-A and MMP-9. On the other hand, they responded differently to a short term exposure to dabrafenib, which induced a further increase of VEGF-A and MMP-9 secretion and of ECM invasion in A375R cells, whereas it did not affect these cellular processes in SK-Mel28R cells. Noteworthy, the basal levels of VEGF-A and MMP-9 secretion in SK-Mel28R cells were about 5-fold and 2-fold higher, respectively, than those detected in A375R cells. Moreover, we previously demonstrated that in A375R cells, dabrafenib-induced stimulation of ECM invasion was dependent on drug-mediated up-regulation of VEGF-A secretion [31]. The high amount of VEGF-A and MMP-9 produced by SK-Mel28R cells themselves, could explain why the release of these cytokines did not increase when the cells were exposed to dabrafenib and why no further stimulation of ECM invasion was induced by either exogenous VEGF-A or dabrafenib treatment.

The increased invasiveness of A375R and SK-Mel28R cells does not appear to be dependent on up-regulation of PTTG1 expression, as both cell lines expressed PTTG1 at levels comparable to those of their matched drug-sensitive counterparts. Nevertheless, the invasive capacity of A375R and SK-mel28R cells was markedly reduced by *PTTG1* knock-down. Moreover, although dabrafenib still stimulated invasiveness in *PTTG1*-silenced A375R cells, ECM invasion by those cells was significantly reduced as compared to that of drug-treated PTTG1-expressing cells. In the clinical setting, melanoma patients developing resistance to BRAFi frequently continue treatment beyond progression. Indeed, it has been demonstrated that prolonging BRAFi therapy beyond RECIST disease progression can provide a clinical benefit [50, 51]. Our findings suggest that targeting PTTG1 in combination with dabrafenib therapy could provide a better disease control, being the impairment of PTTG1 expression able to reduce cell proliferation, restrain the highly invasive behavior associated with acquired resistance to dabrafenib and to counteract possible stimulating effects of the drug on melanoma cell metastatic potential. In this regard, our data show that treatment with the CDK4/6 inhibitor LEE011 at the concentration of 4 μM, which corresponds approximately to the mean value of the peak plasma concentration observed in patients treated with the drug at the dose of 600 mg/day 3-weeks-on/1-week-off [52] was able to reduce PTTG1 levels in A375R cells. Notably, exposure to 4 μM LEE011 caused a significant inhibition of A375R cell proliferation that was maintained also when the CDK4/6 inhibitor was given in combination with dabrafenib. Moreover, although 4 μM LEE011 did not affect A375R basal invasiveness, it abrogated stimulation of ECM invasion induced by dabrafenib in this cell line.

The effects of LEE011 on proliferation and invasiveness of A375R cells were even more pronounced when the drug was used at the concentration of 16 μM, which abrogated PTTG1 expression. Indeed, 16 μM LEE011 not only was more effective than 4 μM LEE011 in reducing A375R cell proliferation, but markedly inhibited basal invasiveness of these cells. Moreover, although the invasive capacity of A375R cells treated with dabrafenib plus 16 μM LEE011 was higher than that displayed by the cells treated with the CDK4/6 inhibitor alone, it still remained lower than that of DMSO-treated cells. These results suggest that inhibition of PTTG1 expression induced by LEE011 contributed in part to drug-induced impairment of cell proliferation and invasiveness. Accordingly, comparable reduction of cell growth and ECM invasion was observed in control and *PTTG1*-silenced A375R cells upon treatment with 16 μM LEE011.

CDK4/6 inhibitors represent promising candidates for cancer therapy (reviewed in [53–55]), since alterations in the cyclin D-CDK4/6-p16^INK4A^-Rb pathway occur frequently in various type of tumors, including the majority of melanomas, and promote continued growth. Actually, palbociclib and LEE011 have been approved by FDA for breast cancer treatment. Moreover, the two drugs and other CDK4/6 inhibitors are under clinical investigation as single agents or in combination therapy in a range of tumors (www.clinicaltrial.gov).

Although CDK4/6 inhibitors can reduce melanoma cells growth both *in vitro* and in animal models, the effects of these drugs appear to be mainly cytostatic [53, 55]. Therefore, monotherapy with CDK4/6 inhibitors is not considered an effective treatment modality in melanoma. On the other hand, synergistic or additive antiproliferative effects of concomitant inhibition of CDK4/6 and the MAPK pathway have been shown in melanoma cells [53, 55, 56]. Moreover, Yadav *et al.* [56] demonstrated that the CDK4/6 inhibitor LY2835219 was highly effective in suppressing, both *in vitro* and *in vivo*, the growth of melanoma cells with acquired resistance to the BRAFi vemurafenib. Our results are consistent with those previous findings. Moreover, they demonstrate that CDK4/6 inhibitors can also reduce invasiveness of BRAFi-resistant cells and counteract possible stimulating effects of BRAFi on the invasive capacity of those cells, thus providing further experimental support to therapeutic strategies combining inhibitors of the MAPK pathway with drugs selectively targeting CDK4/6.

In the present study, we also investigated whether down-regulation of MMP-9 secretion could be involved in the impairment of ECM invasion caused by *PTTG1* knock-down in dabrafenib-resistant cells. Actually, MMP-9 plays an important role in melanoma invasion [35, 36] and PTTG1 has been shown to positively regulate the expression levels of MMP-9 [21, 33, 34].

Our data strongly support the involvement of MMP-9 down-regulation in the effects of *PTTG1* silencing on invasiveness of dabrafenib-resistant cells. Indeed, *PTTG1* knock-down in A375R cells before exposure to either DMSO or dabrafenib significantly reduced MMP-9 secretion. Moreover, the invasive capacity of control and drug-treated cells resulted inhibited when assayed in the presence of an anti-MMP-9 mAb. Finally, in both DMSO- and dabrafenib-treated cells, *PTTG1* silencing, MMP-9 inhibition or their combination induced comparable levels of inhibition of ECM invasion.

### Changes of PTTG1 plasma levels in melanoma patients subjected to targeted therapy

The PTTG1 protein has been reported to be detectable in plasma of healthy subjects and patients with colorectal neuroendocrine tumor [37]. Therefore, the finding that upon exposure to dabrafenib PTTG1 expression was markedly impaired only in melanoma cells sensitive to the drug and that this molecular event contributed to dabrafenib-induced inhibition of cell proliferation and invasiveness, prompt us to conduct an exploratory study to assess whether the PTTG1 protein could be detected also in plasma of melanoma patients and whether changes in circulating PTTG1 levels occurred during targeted therapy and were related to patient response.

Even if limited by the small number of patients examined, our preliminary investigation provides interesting findings. Indeed, it demonstrates that the PTTG1 protein was present in plasma of a considerable proportion of melanoma patients even though in different amounts. The study also shows that after two months of therapy, a decrease of circulating PTTG1 occurred in about 70% of responder patients with detectable baseline levels of the protein, and that in 60% of these patients PTTG1 levels rose again at disease progression. In contrast, in only 1 patient among the non-responder group a reduction of plasma PTTG1 was observed at T2. Although it remains to be ascertain whether the decrease of circulating PTTG1 in responder patients simply reflects the reduction of tumor burden or is also due to drug-induced down-regulation of PTTG1 expression in melanoma cells, these findings are consistent with and reinforce our *in vitro* results. However, it must be pointed out that when responder patients were considered in their whole number, the T2-TP but not the T0-T2 PTTG1 comparison reached the statistical significance, most probably as a result of the low number of patients examined. Therefore, although our data suggest that monitoring circulating PTTG1 during therapy with BRAFi or BRAFi plus MEKi might provide useful information about patient response, this need to be confirmed in a larger study, that was beyond the scope of the present investigation.

## CONCLUSIONS

Our results provide evidence that PTTG1 is involved in the positive regulation of melanoma cell proliferation and invasiveness. They also demonstrate that in dabrafenib-sensitive cells, inhibition of cell growth and ECM invasion caused by this drug occur at least in part, through down-regulation of PTTG1 expression. More important, our study shows that in dabrafenib-resistant cells, inhibition of PTTG1 expression efficiently impairs proliferation and invasiveness, suggesting that in patients progressing on dabrafenib therapy, PTTG1 targeting, alone or in combination with BRAFi, could represent a useful strategy to control tumor growth and metastatic spreading. Finally, we present preliminary evidences that circulating PTTG1 might represent a novel biomarker to monitor patient response to targeted therapy.

## MATERIALS AND METHODS

### Cell cultures

The human melanoma cell lines A375 and SK-Mel28 were purchased from the European Collection of Cell Cultures (Salisbury, UK) and American Type Culture Collection (ATCC, Manassas, VA) respectively. The cells were cultured in BioWhittaker™ RPMI-1640 medium (LONZA, Verviers, Belgium) supplemented with 10% fetal bovine serum (Sigma-Aldrich, St. Louis, MO), 2 mM BioWhittaker™ L-glutamine (LONZA), and 50 µg/ml BioWhittaker™ gentamicin (LONZA) (hereafter referred to as complete medium, CM). The dabrafenib-resistant A375R cell line, generated in our laboratory, has been previously described [31]. The dabrafenib-resistant SK-Mel28R cell line was generated in the present study by growing the parental SK-Mel28 cells in gradually increasing concentrations of dabrafenib (from 1 nM up to 1.5 µM), as previously reported for the generation of the A375R cell line. A375R and SK-Mel28R cell lines were maintained in CM supplemented with 1.5 µM dabrafenib.

### Drugs, chemicals and antibodies for Western blot analysis

Dabrafenib (GSK2118436A) and LEE011 (Active Biochem, Hong Kong) were dissolved in DMSO (Sigma-Aldrich) at a final concentration of 1.92 mM and 16 mM, respectively. Drugs were stored as stock solutions at –80°C and diluted in CM just before use.

3-(4,5-dimethylthiazol-2-yl)-2,5-diphenyltetrazolium bromide (MTT) was purchased from Sigma-Aldrich, dissolved at a concentration of 5 mg/ml in GIBCO™ Phosphate-Buffered Saline (PBS) (Invitrogen, Thermo Fisher Scientific, Waltham, MA) and stored at 4°C.

Mouse monoclonal antibody (mAb) against PTTG1 (DCS-280) and rabbit polyclonal antibody against human β-tubulin (sc-9104) were purchased from Santa Cruz Biotechnology, Inc., (Santa Cruz, CA).

Reagents for sodium dodecyl sulphate (SDS)-polyacrylamide gel electrophoresis were all purchased from Bio-Rad Laboratories, Inc. (Hercules, CA).

### Chemosensitivity assay

Melanoma cells were suspended in CM, seeded (50 µl/well) into BD Falcon™ 96-well plates (BD Biosciences, Bedford, MA) and allowed to adhere at 37°C in a 5% CO_2_ atmosphere for 18 h. Graded amounts of dabrafenib were then added to the cells in 50 µl of CM. As a control, melanoma cells were treated with DMSO alone. The plates were incubated at 37°C for five days and cell proliferation was then evaluated by the MTT assay, as previously described [57]. Three replica wells were used for each group. Drug concentration producing 50% inhibition of cell growth (i.e. IC_50_) was calculated on the regression line in which absorbance values at 595 nm were plotted against the logarithm of drug concentration.

### Transient transfection with siRNA and dabrafenib treatment of the transfected cells

Oligonucleotide siRNA targeting *PTTG1* (siPTTG1) and All Star Negative Control (siCTRL) were obtained from Ambion (Austin, TX) and Qiagen (Hilden, Germany), respectively. Transfection was performed using Lipofectamine™ RNAiMAX reagent (Invitrogen Corporation, Carlsbad, CA) according to the manufacturer’s protocol.

For proliferation assays, melanoma cells were suspended in CM without antibiotics, seeded (100 µl/well) into BD Falcon™ 96-well plates (BD Biosciences) and allowed to adhere at 37°C in a 5% CO_2_ atmosphere for 18 h. The cells were then transfected with 10 nM siPTTG1 or siCTRL and analyzed for proliferation three and six days after transfection using the MTT assay. Three replica wells were used for each group.

For chemosensitivity assays, cells were suspended in CM without antibiotics, seeded into 96-well plates (Falcon), and allowed to adhere at 37°C for 18 h. The cells were then transfected with 10 nM siPTTG1 or siCTRL. After 24 h of incubation, the cells were exposed to DMSO alone or to graded concentrations of dabrafenib (range: 0.048–6.25 nM for A375; 0.195–25 nM for SK-Mel28, 50–6400 nM for A375R and SK-Mel28R). The plates were incubated at 37°C for three or five days and cell growth was then evaluated by the MTT assay. Three replica wells were used for each group.

For Western blot analysis, invasion assay and evaluation of MMP-9 secretion, melanoma cells were suspended in CM without antibiotics, seeded into BD Falcon™ 6 cm-dishes (BD Biosciences), allowed to adhere at 37°C for 18 h, and then transfected with 10 nM siPTTG1 or siCTRL. After six days of incubation, the cells were processed for Western blot analysis. Alternatively, 24 h after transfection, 100 nM dabrafenib or DMSO was added to the cultures and the dishes incubated at 37°C for additional 48 h. At the end of the incubation period, culture supernatants were recovered for MMP-9 determination and the cells were detached, counted and processed for Western blot analysis or tested for their ability to invade the ECM *in vitro*.

### Transient transfection with a *PTTG1* expression vector and dabrafenib treatment of the transfected cells

The pCMV6-Entry expression vector encoding a FLAG-tagged PTTG1 protein (CMV-PTTG1) and the empty vector (CMV-EV) were purchased from OriGene Technologies, Inc. (Rockville, MD).

For proliferation assays, melanoma cells were suspended in CM without antibiotics, seeded (100 µl/well) into BD Falcon™ 96-well plates (BD Biosciences) and allowed to adhere at 37°C in a 5% CO_2_ atmosphere for 18 h. The cells were then transfected with 5 µg CMV-PTTG1 or CMV-EV using Lipofectamine™ 2000 (Invitrogen), according to the manufacturer’s protocol. After 24 h of incubation, medium was replaced with fresh CM without antibiotics and the cells were analyzed for proliferation five days later using the MTT assay. Three replica wells were used for each group.

For chemosensitivity assays, cells were suspended in CM without antibiotics, seeded into 96-well plates (Falcon), and allowed to adhere at 37°C for 18 h. The cells were then transfected with 5 µg CMV-PTTG1 or CMV-EV. After 24 h of incubation medium was replaced with fresh CM containing DMSO alone or graded concentrations of dabrafenib (range: 0.048–6.25 nM). The plates were incubated at 37°C for five days and cell growth was then evaluated by the MTT assay. Three replica wells were used for each group.

For Western blot analysis and invasion assay, A375 cells were suspended in CM without antibiotics, seeded into BD Falcon™ 6 cm-dishes (BD Biosciences), allowed to adhere at 37°C for 18 h, and then transfected with 5 µg CMV-PTTG1 or CMV-EV. After 24 h of incubation, medium was replaced with fresh CM and the dishes were incubated at 37°C for additional five days. A375 cells were then processed for Western blot analysis. Alternatively, 24 h after transfection medium was replaced with fresh CM containing 100 nM dabrafenib or DMSO and the dishes were incubated at 37°C for additional 48 h. Melanoma cells were then processed for Western blot analysis or tested for their ability to invade the ECM *in vitro*.

### Treatment of A375R with LEE011 alone or in combination with dabrafenib

A375R cells were suspended in CM without antibiotics, seeded into BD Falcon™ 6 cm-dishes (BD Biosciences), and allowed to adhere at 37°C for 18 h. Thereafter, the cells were exposed to LEE011 (4 μM or 16 μM), dabrafenib (100 nM) or a combination of LEE011 (4 μM or 16 μM) plus dabrafenib for 48 h. At the end of the incubation period, the cells were recovered, counted in a hemocytometer to determine cell proliferation, and then assayed for PTTG1 expression and ability to invade the ECM *in vitro*. Control groups were treated with DMSO alone. A375R were also transfected with 10 nM siPTTG1 or siCTRL and 24 h later incubated with DMSO or 16 μM LEE011. After 48 h of culture the cells were recovered counted in a hemocytometer to determine cell proliferation and assayed for ECM invasion.

### Western blot analysis

Melanoma cells were recovered from culture, washed and total cellular extracts were prepared as described previously [57]. Fifteen μg of proteins per sample were run on 12% SDS-polyacrylamide gels, transferred to nitrocellulose membranes (Amersham Biosciences, Buckinghamshire, UK) and blocked with 5% non-fat milk in Tris-buffered saline supplemented with 0.1% Tween 20 (TBST-milk) for 1 h at room temperature. The membranes were then incubated in TBST-milk overnight at 4°C with primarys antibodies at the following dilutions: anti-β-tubulin 1:1000 and anti-PTTG1 1:500. The anti-β-tubulin antibody was used as an internal standard for loading. Immunodetection was carried out using appropriate horseradish peroxidase-linked secondary antibodies and developed with Clarity™ Western ECL Substrate (Bio-Rad Laboratories, Inc.). Where indicated, films were scanned on a GS-710 Calibrated Imaging Densitometer and analyzed by means of Quantity One Software Version 4.1.1 (Bio-Rad Laboratories, Inc.).

### Invasion assay in Boyden chambers

This assay was performed as previously described [58]. Briefly, melanoma cells were removed from culture, washed, suspended in invasion medium (1 µg/ml heparin/0.1% bovine serum albumin in RPMI-1640) and loaded (1 × 10^5^ cells) into the upper compartment of Boyden chambers equipped with 8 µm pore diameter polycarbonate filters (Nuclepore, Whatman Inc., Clifton, NJ) coated with 20 µg of BD Matrigel™ Basement Membrane Matrix (BD Biosciences). Invasion medium or, where indicated, invasion medium containing 20 ng/ml VEGF-A (ImmunoTools, Friesoythe, Germany) was added to the lower compartment of the chambers. For each experimental condition, three Boyden chambers were set up. After incubation of the Boyden chambers at 37°C in a 5% CO_2_ atmosphere for 4 h, the filters were removed from the chambers and the cells were fixed in ethanol for 5 min and stained in 0.5% crystal violet for 15 min. The cells from the upper surface of the filter were removed by wiping with a cotton swab and the migrated cells, attached to the lower surface of the filters, were counted under the microscope. Twelve microscopic fields (x200 magnification), randomly selected on triplicate filters, were scored for each experimental condition.

In a set of experiments, invasion assays were performed in the presence of 5 µg/ml of an anti-human MMP-9 mAb (Ab-3, 56-2A4; Calbiochem) or the corresponding control IgG immunoglobulin (MAB002; R&D Systems, Minneapolis, MN). Melanoma cells were pre-incubated with the mAb for 30 min at room temperature in a rotating wheel. The cells were then loaded in the Boyden chambers without removing the mAb.

### Evaluation of MMP-9 secretion

Culture supernatants were collected, centrifuged at 600 × g for 10 min to remove cells in suspension and debris, and frozen at –80°C until use. Cells were detached with a solution of 1.5 mM EDTA in PBS and counted to determine the total number of cells in the culture. Culture supernatants were concentrated at least ten-fold in Centriplus concentrators (Amicon, Beverly, MA). The amount of active MMP-9 in the culture supernatants was then determined using the Quantikine ELISA Human MMP-9 Immunoassay (R&D Systems) according to the manufacturer’s protocol, and calibrated against a standard curve. The amount MMP-9 was normalized to the number of total cells counted in each culture at the time of supernatant collection.

### Patients

Plasma levels of PTTG1 protein were determined in 22 patients with BRAF^V600^-mutant metastatic cutaneous melanoma consecutively treated with dabrafenib, vemurafenib or dabrafenib plus trametinib at Istituto Dermopatico dell’Immacolata (IDI)-IRCCS and from whom peripheral blood samples had been sequentially collected before therapy commencement and up to disease progression. Baseline evaluation included medical history, physical examination, and radiologic tumor assessment with computer tomography or positron emission tomography scans. Dabrafenib (Tafinlar^®^) was given at the dose of 150 mg BID, vemurafenib (Zelboraf^®^) at the dose of 960 mg BID and dabrafenib plus trametinib (Mekinist^®^) at the dose of 150 mg BID and 2 mg/die, respectively. All patients underwent physical examination and assessment of biochemical parameters monthly, whereas tumor response was determined with CT every three months. Tumor response was classified as complete response, partial response, stable disease or progressive disease according to RECIST 1.1 criteria [59]. Time to treatment failure was defined as the time from the start of therapy to the first observation of disease progression per RECIST 1.1. The study was conducted in accordance with Good Clinical Practice Guidelines and the Declaration of Helsinki. The study was also approved by the IDI-IRCCS Ethics Committee (ID #407/1, 2013 and #407/2, 2016) and a written informed consent was obtained from all patients.

### Plasma preparation and PTTG1 evaluation

Blood was collected into BD Vacutainer^®^ tubes (#367704, BD Biosciences, Plymouth, UK), double centrifuged at 1,200 × g for 10 minutes at 4 C°, and the plasma stored at –80°C within 2 h from collection.

PTTG1 amount in plasma samples was determined using Human Securin (PTTG1) ELISA Kit (MyBioSource, Inc., San Diego, CA) according to the manufacturer’s instructions. All determinations were performed in duplicate. A value of 0.1 ng/mL was the lower limit of PTTG1 detection of the ELISA kit. Therefore, for data analysis, assay values under the lower limit of ELISA detection were replaced with 0.01 ng/mL as previously described [60].

### Statistical analysis

Statistical significance of the differences among experimental groups was assessed using unpaired two-side Student’s *t-*test, whereas statistical significance of the differences between PTTG1 plasma levels at different time points (T2 *versus* T0 and TP *versus* T2) in each melanoma patient was assessed using paired two-side Student’s *t*-test. Significance was set at *P* < 0.05.

Plasma levels of PTTG1 were also reported as medians and Interquartile Range (IQR) and were analysed using nonparametric procedures. The Mann-Whitney *U*-test was used to compare between-group differences, while the Wilcoxon matched-pairs signed-rank test was used to evaluate before-after differences. Nonparametric analyses were conducted using STATA11 (Stata Corp. LP, College Station, TX).

## SUPPLEMENTARY MATERIALS FIGURE



## Abbreviations

BRAFi: BRAF inhibitor
CM: complete medium
DMSO: dimethyl sulfoxide
ECM: extracellular matrix
IC: inhibitory concentration
MAPK: mitogen-activated protein kinase
MEKi: MEK inhibitor
MMP: matrix metalloproteinase
MTT: 3-(4,5-dimethylthiazol-2-yl)-2,5-diphenyltetrazolium bromide
*PTTG1*: pituitary tumor transforming gene 1
siRNA: small interfering RNA
PBS: phosphate buffered saline
SDS: sodium dodecyl sulphate
SEM: standard error of the mean
TBST: Tris-buffered saline/Tween 20
VEGF: vascular endothelial growth factor
*vs*: *versus*
