# Supplementary material for: Targeting the PTTG1 oncogene impairs proliferation and invasiveness of melanoma cells sensitive or with acquired resistance to the BRAF inhibitor dabrafenib
Source: Oncotarget. 2017 Dec 9;8(69):113472–93. doi: 10.18632/oncotarget.23052 (PMC5768340; doi:10.18632/oncotarget.23052)
Supplement: Supplementary file 1 [file oncotarget-08-113472-s001.pdf]

Targeting the *PTTG1* oncogene impairs proliferation and invasiveness of melanoma cells sensitive or with acquired resistance to the BRAF inhibitor dabrafenib

SUPPLEMENTARY MATERIALS

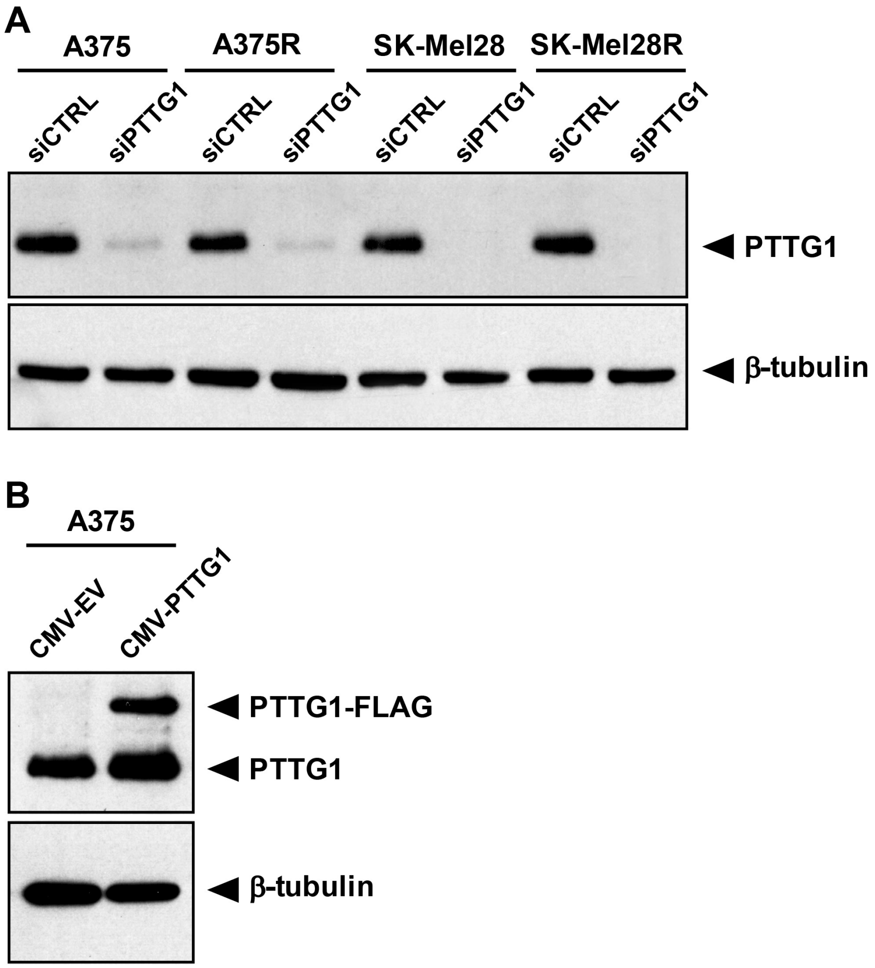

Supplementary Figure 1: Evaluation of PTTG1 protein levels after six days of *PTTG1* silencing or over-expression. (**A**) Melanoma cells were transiently transfected with siPTTG1 or siCTRL and after six days of culture, cell extracts were prepared and analyzed by immunoblotting using antibodies against PTTG1 or against β-tubulin. The results are representative of three independent experiments. (**B**) A375 cells were transiently transfected with CMV-PTTG1 or CMV-EV vectors. After six days, cell lysates were analyzed by immunoblotting using antibodies against PTTG1 or against β-tubulin. The results are representative of three independent experiments.
